# Integrated Phenotypic, Cytotypic, and Microsatellite Diversity Analysis of Wild-Growing/Naturalized Ber (*Ziziphus mauritiana* Lam.) Across Pakistan: Implications for Germplasm Conservation and Breeding

**DOI:** 10.3390/plants15131974

**Published:** 2026-06-26

**Authors:** Mian Fazli Basit, Nadeem Bhanbhro, Fazli Rahim, Jian Huang

**Affiliations:** 1 Key Laboratory of National Forestry and Grassland Administration on Silviculture in Loess Plateau, College of Forestry, Northwest A&F University, No. 3 Taicheng Road, Yangling 712100, China; mianbasit@nwafu.edu.cn; 2 Sanya Institute, Hainan Academy of Agricultural Sciences, Sanya 572000, China; 3 Institute of Crop Sciences, Chinese Academy of Agricultural Sciences, Beijing 100081, China; 4 National Nanfan Research Institute, Chinese Academy of Agricultural Sciences, Sanya 572000, China; 5 Department of Botany, University of Buner, Swari 19290, Khyber Pakhtunkhwa, Pakistan

**Keywords:** *Ziziphus mauritiana*, genetic diversity, polyploidy, flow cytometry, population structure, SSR markers, germplasm conservation, Pakistan

## Abstract

*Ziziphus mauritiana* Lam. (ber or Indian jujube) is a stress-tolerant dryland fruit tree valued for its nutritious fruit and ability to grow on marginal land. However, the phenotypic, cytotypic and genetic structure of its wild-growing/naturalized germplasm in Pakistan remains poorly characterized. This study provides an integrated assessment of phenotypic, cytotypic and simple-sequence-repeat (SSR) diversity in 100 wild-growing/naturalized accessions collected from Khyber Pakhtunkhwa, Punjab and Sindh to establish a baseline for conservation and germplasm management. We recorded 37 morphological and biochemical traits, estimated ploidy levels by flow cytometry (using diploid *Z. jujuba* ‘Dongzao’ as a reference), and genotyped a representative subset of 60 accessions with 14 SSR markers scored as a binary presence/absence matrix. Substantial phenotypic variation was observed, especially in canopy architecture, leaf traits and stone-related characteristics; fruit quality traits (total soluble solids, vitamin C, and acidity) varied within a narrower range. Province explained only a modest proportion of phenotypic variation (PERMANOVA R^2^ = 0.059–0.109; *p* < 0.01), with extensive overlap among regions. Flow cytometry revealed polyploid diversity: hexaploid (2*n* = 6x = 72) accessions dominated (46.7%), followed by octoploid (2*n* = 8x = 96; 31.7%) and tetraploid (2*n* = 4x = 48; 21.7%) cytotypes. SSR analysis showed moderate within-province diversity (Nei’s H ≈ 0.51) but negligible genetic differentiation among provinces (R^2^ = 0.030; *p* = 0.60; Φ ≈ −0.011), indicating weak geographic structuring. Wild-growing/naturalized *Z. mauritiana* in Pakistan forms a diverse, weakly structured gene pool in which most variation occurs within rather than among provinces. Sampling for conservation and germplasm management should, therefore, prioritize phenotypic distinctiveness, cytotype representation and ecological context rather than geographic origin alone. Experimental validation of any adaptive or agronomic advantages of particular cytotypes is needed before breeding recommendations can be made.

## 1. Introduction

*Ziziphus mauritiana* Lam. (Ber or Indian jujube) is a resilient fruit tree well adapted to tropical and subtropical drylands. It is considered one of the most stress-tolerant members of the genus *Ziziphus* [1]. The species grows reliably on marginal soils and under limited irrigation, thereby becoming an important crop for low-input farming and dryland livelihoods. Its fruits are valued for their marketability, flavor and nutritional quality, particularly because they are rich in vitamin C, minerals, dietary fiber, carbohydrates, and other essential nutrients [2,3,4,5,6]. *Z. mauritiana* is an extensively distributed species in Pakistan, in both managed environments (cultivated) and in unmanaged habitats, along the sides of roads, foothills, arid plains, and rangelands. The unmanaged populations are often termed wild-growing/naturalized, although many may more accurately represent naturalized, self-sustaining stands.

Despite its ecological and economic importance, the diversity of wild-growing/naturalized *Z. mauritiana* in Pakistan remains insufficiently characterized at the national scale. Previous studies have documented variation within Pakistani germplasm and have highlighted its potential for conservation and improvement [7]; however, the available evidence remains fragmented across locations and trait sets. This limits the ability to identify representative germplasm, define conservation priorities, and select parental material for breeding. Integrated evaluation is particularly important because phenotypic performance alone may not fully reflect underlying genetic diversity, especially in long-lived, outcrossing, and environmentally plastic fruit trees [8].

A second gap concerns the cytogenetic composition of *Z. mauritiana.* Polyploidy is common in the genus *Ziziphus*, which has a basic chromosome number of x = 12. *Z. mauritiana* is recognized for its polyploid nature, with cytotypes ranging from tetraploid (2*n* = 4x = 48) to higher ploidy levels (e.g., hexaploid and octoploid) reported in different regions [5]. Recent high-quality genomic studies also support an autotetraploid genome structure and provide an important framework for linking domestication processes and fruit quality traits to genome evolution [9,10,11]. However, the cytotype composition of wild-growing/naturalized *Z. mauritiana* populations in Pakistan has not yet been investigated, leaving the national distribution of ploidy levels unknown. This represents an important gap because cytotype variation can influence morphology, fertility, adaptation and breeding compatibility. Without this information, interpreting trait variation and developing effective breeding or conservation strategies becomes difficult, as true biological diversity may be overlooked or redundantly sampled.

Molecular markers provide a complementary approach for resolving genetic diversity and population structure. Simple sequence repeat (SSR) markers are particularly useful because of their polymorphism, reproducibility, co-dominant inheritance and transferability across related taxa [9,12,13]. SSRs have been applied successfully in *Ziziphus* to distinguish cultivars, assess genetic relationships, and evaluate diversity across wild and cultivated germplasm [9,14]. However, interpreting SSR profiles in polyploid material requires careful scoring, particularly when allele dosage cannot be reliably inferred. In such cases, presence/absence-based marker matrices can provide a practical framework for estimating diversity and comparing genetic structure among accessions.

An integrated phenotypic, cytotypic and genetic approach is needed in polyploid fruit trees because no single method is sufficient on its own. Phenotypic traits can be shaped by the environment and do not always reflect genetic differences. Molecular markers show genetic relationships but are harder to interpret in polyploids, where allele dosage cannot be scored reliably. Ploidy analysis identifies different cytotypes that markers and morphology may otherwise miss. Using the three together, therefore, gives a clearer and more complete picture of diversity than any one alone. This is supported by recent genomic studies of *Z. mauritiana*, which show that polyploidy and domestication have together shaped its genome and fruit traits [11,15].

In this study, we provide an integrated characterization of wild-growing/naturalized *Z. mauritiana* genetic resources across Pakistan, combining standardized phenotyping with flow-cytometry ploidy estimation and SSR profiling. We hypothesized that (i) these populations harbor substantial phenotypic and cytotypic diversity but only weak geographic structuring and (ii) phenotypic variation is driven more by trait architecture than by province of origin. We accordingly expected to detect multiple cytotypes, broad within-region phenotypic variation and low among-province genetic differentiation. The novelty of this study lies in delivering the first cytotype report for Pakistani *Z. mauritiana* and in linking ploidy, phenotype and SSR-based diversity within a single framework, thereby establishing a practical baseline for germplasm management, conservation prioritization and parent selection.

## 2. Materials and Methods

### 2.1. Sample Collection

**Figure 1:**
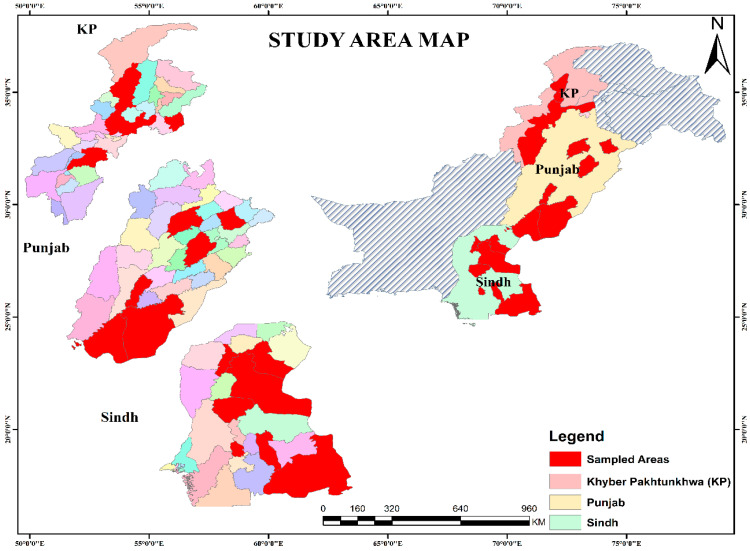
Study area map showing the sampled districts across Khyber Pakhtunkhwa, Punjab, and Sindh, Pakistan. Provinces are color-coded; sampling districts are marked. A scale bar (km) and north arrow are included. Hatched grey areas indicate regions outside the surveyed provinces.

**Figure 2:**
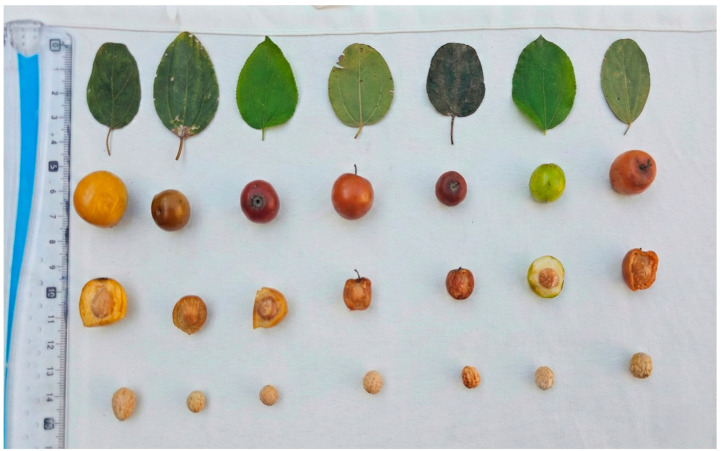
Representative phenotypic variation in wild-growing/naturalized ber (*Ziziphus mauritiana* Lam.) accessions showing differences in leaf morphology, fruit size/shape/color, fruit cross-sections (pulp/stone) and stones. A ruler is included for scale.

A total of 100 wild-growing/naturalized accessions of *Ziziphus mauritiana* Lam. were collected from Khyber Pakhtunkhwa, Punjab and Sindh, Pakistan, between 2023 and 2025 (Figure 1 and Figure 2). Sampling was conducted using a stratified field-sampling approach to include the major unmanaged habitats in which *Z. mauritiana* occurs in the surveyed regions, including roadsides, mountain foothills, desert margins, arid plains and rangelands (Supplementary Figure S1; Supplementary Table S1). Accessions were distributed across the three provinces and multiple districts to represent broad geographic and habitat coverage. To reduce repeated sampling of the same clone, closely related neighboring individuals, or root-suckering material, sampled trees were spatially separated by at least 1.0–1.5 km where field conditions allowed. Multiple stems from the same crown, clump, or immediately adjacent stand were avoided. Only unmanaged, self-sustaining trees growing outside cultivated orchards and without evidence of active horticultural management were included. For each accession, mature fruits, leaves and stones (hard endocarps containing seeds) were collected randomly in 3 replicates during the fruiting season. Leaves were dried using silica gel enclosed in zipper bags to ensure rapid moisture removal. The stones (hard endocarps containing seeds) were air-dried in paper bags at room temperature. Fruits were processed on the day of harvest to ensure accuracy and consistency in biochemical assays. A total of 37 traits (14 quantitative and 23 qualitative) were studied. A digital caliper (model HT0403-A1; Cingda Industry Co., Ltd., Nanjing, China) was used to measure stone length, stone width, fruit length, leaf length, leaf width and fruit width with an accuracy of 0.10 mm, while an electric weighing balance was used to measure stone weight.

### 2.2. Quantitative Traits

Fourteen quantitative traits were documented for diversity analysis: east–west canopy spread, north–south canopy spread, fruit length, fruit width, leaf width, leaf length, stone length, stone width, stone weight, thorn size, stem girth, acidity, total soluble solids (TSSs; °Brix) and vitamin C (mg 100 g^−1^). Stem girth was measured as the trunk circumference at 4.5 ft aboveground by wrapping a measuring tape around the trunk and reading the value directly [7]. Canopy spread was assessed by measuring east–west and north–south spread using a meter rod, independent of trunk position and based on limb tips [16]. Fruit quality parameters, including total soluble solids and acidity, were determined using a digital refractometer (RX 5000, ATAGO, Tokyo, Japan) and the Horwitz method, respectively [17]. Vitamin C content in fruit flesh was determined using the 2,6-dichlorophenolindophenol dye-titration method described by Ruck [18].

### 2.3. Qualitative Traits

Twenty-three qualitative traits were visually assessed and recorded for each accession using the Ber plant descriptor [19] on coded scales and used for diversity estimation. The recorded qualitative traits included tree growth habit, shoot surface, leaf apex, leaf base, leaf shape, leaf pubescence (lower surface), thorn attachment, thorn arrangement, thorn shape, fruit shape, fruit surface, fruit apex, fruit color, stone apex shape, stone shape, stone surface, leaf color (lower), leaf color (upper), leaf margins, leaf veins (upper), leaf veins (lower), leaf petiole color and foliage density were visually observed for estimation of diversity. All measurements and visual assessments were performed on at least 10 fruits and 10 leaves per accession to ensure representative sampling.

### 2.4. Seed Germination and Seedling Growth

The collected stones were surface-cleaned, soaked in sterile water for 24 h and sown in trays under greenhouse conditions in China (25 ± 2 °C, 60–70% RH, and 16 h light/8 h dark). Watering was maintained near field capacity. After 3 weeks, young leaves from healthy seedlings were harvested for further analysis.

### 2.5. Ploidy Level Analysis

Ploidy levels were estimated by flow cytometry using young leaf tissue from greenhouse-grown seedlings. Fresh young leaves were finely chopped in ice-cold WPB nuclei isolation buffer, and the nuclei suspension was passed through a 30–50 µm mesh filter. The filtrate was treated with RNase A at 50 µg mL^−1^ for 10 min at room temperature in the dark, followed by staining with propidium iodide (PI; 50 µg mL^−1^) for 15–20 min under dark conditions. The prepared nuclei suspensions were analyzed using a BD Accuri C6 flow cytometer. Throughout the procedure, the samples were kept on ice and protected from light.

The diploid Chinese jujube (*Ziziphus jujuba* Mill.) cultivar ‘Dongzao’ (2*n* = 2x = 24) was used as an external 2x reference standard for estimating relative nuclear DNA content [9]. It was analyzed both separately and in mixed preparations with selected accessions to establish the diploid G0/G1 peak position and verify sample peak positions. PI fluorescence was recorded using the PE-A/FL2-A area parameter. Intact nuclei were selected by excluding debris and poorly resolved events, and the G0/G1 peak was identified from the PE-A fluorescence histogram.

Each accession was processed in duplicate to confirm reproducibility. Only histograms with distinct G0/G1 peaks, reproducible peak positions and peak coefficients of variation below 5% were retained for cytotype assignment. Cytotype assignment was based on the ratio of the sample G0/G1 PE-A mean fluorescence to the Dongzao G0/G1 PE-A mean fluorescence, calculated as follows: Ratio = Sample G0/G1 PE-A mean fluorescence/Dongzao control G0/G1 PE-A mean fluorescence. Accessions with fluorescence ratio classes close to 2.0, 3.0, and 4.0 relative to the diploid Dongzao reference were assigned as tetraploid (4x), hexaploid (6x), and octoploid (8x), respectively. Representative histograms for the reference standard, mixed reference/sample run and inferred cytotypes are discussed in the results.

Ratio=(G_0/G_1 Sample)/(G_0/G_1 Control mean)

### 2.6. SSR Genotyping and Binary Marker Scoring

A province-balanced representative subset of 60 accessions was selected from the 100 sampled accessions for SSR analysis, comprising 20 accessions from each province. This subset was designed to retain geographic, district-level, habitat, phenotypic and cytotypic coverage of the full collection while ensuring high-quality DNA for PCR amplification. Accessions were selected to include representatives from each province and major sampled habitat type, while avoiding spatially adjacent individuals and phenotypically redundant neighboring trees. Therefore, the SSR subset was intended to represent the breadth of the sampled germplasm while reducing redundancy, rather than representing a single locality, habitat, or phenotype class. Total genomic DNA was then extracted from silica-dried leaves using a commercial plant DNA Kit (TIANGEN Biotech (Beijing) Co., Ltd., Beijing, China). DNA quality and concentration were assessed using NanoDrop. The fourteen nuclear SSR primer pairs previously reported by Liang et al. [9] were used for genotyping. Primer information is provided in Supplementary Table S2. PCR amplification was performed in a total reaction volume of 10 µL, containing 1 µL of template DNA, 5 µL of 2× GoldStar Best MasterMix (Kangwei, Beijing, China), 0.5 µL of the forward primer, 0.5 µL of the reverse primer, and 3 µL of nuclease-free water. Cycling profile: 95 °C for 10 min; 35 cycles at 94 °C for 30 s, 50–60 °C for 30 s (primer-specific Ta), and 72 °C for 30 s; and a final extension at 72 °C for 5 min. PCR products were separated on 1.2% agarose and visualized under UV to confirm amplification efficiency. Fragment patterns were scored in GelAnalyzer v23.1.1. For each primer pair, distinct fragment-size classes detected on agarose were recorded as band classes rather than as allelic states. Because this study included polyploid cytotypes, and allele dosage could not be inferred reliably from agarose profiles, fragments were scored as binary band classes (presence = 1; absence = 0) rather than as dosage-resolved codominant SSR genotypes. This multilocus band presence matrix (1/0) was used to estimate SSR band pattern diversity. Within this binary framework, minor band frequency (the frequency of the less common state at a locus), polymorphic information content (PIC) and Nei’s gene diversity (H) were computed from observed band frequencies under a dominant-marker model; H is, therefore, interpreted as band pattern diversity rather than as expected heterozygosity derived from codominant genotypes. The same matrix was used to calculate Jaccard dissimilarities for ordination (PCoA), clustering (UPGMA), PERMANOVA and AMOVA. This binary approach is appropriate for comparing polyploid accessions when allele dosage cannot be resolved, but it has lower resolution than capillary electrophoresis or sequencing-based genotyping, and the genetic results should be interpreted accordingly.

### 2.7. Statistical Analysis

All analyses were performed in R version 4.5.1 (tidyverse, FactoMineR, vegan, ade4, pegas, adegenet, and poppr). Quantitative traits were standardized (mean-centered; unit variance) before PCA and clustering. One-way ANOVA with Tukey’s HSD tested province effects, with residual normality checked by Shapiro–Wilk. Pearson and Spearman correlations were used for quantitative and ordinal traits and Cramér’s V for categorical descriptors. MCA of qualitative traits was performed in FactoMineR, with low-frequency categories retained but interpreted cautiously. Hierarchical clustering used Euclidean/Ward.D2 (quantitative) and Gower/Ward (combined) distances. Differentiation among provinces was tested by PERMANOVA (vegan::adonis2; 999 permutations), with dispersion homogeneity confirmed by betadisper. For the binary SSR band presence matrix, minor band frequency, PIC and Nei’s gene diversity (H) were calculated from band frequencies under a dominant-marker framework, with H interpreted as band pattern diversity; genetic distances were binary Jaccard, and differentiation was assessed by PERMANOVA, AMOVA (ade4) and the fixation index (Φ).

Mixed models and Mantel tests were considered but were not used as primary analyses. Mixed models were not applied because the survey did not include a balanced nested site structure suitable for estimating site-level or habitat-level random effects. Mantel tests were also not performed because complete georeferenced environmental distance matrices were unavailable for all accessions. Therefore, ordination and multivariate tests were interpreted cautiously. PCA, MCA, PCoA and PERMANOVA were used to summarize major multivariate patterns, but modest explained variance values were interpreted as exploratory evidence of multidimensional variation rather than as strong geographic separation.

## 3. Results

### 3.1. Quantitative Trait Analysis

Descriptive statistics for the 14 quantitative traits are presented in Table 1. The 100 wild-growing/naturalized *Ziziphus mauritiana* accessions showed substantial variation across vegetative, fruit, stone, and biochemical traits. The greatest dispersion was observed for stem girth, stone weight, stone length, stone width, canopy spread and leaf dimensions, indicating marked structural and stone-related diversity among accessions. In contrast, fruit biochemical traits, particularly vitamin C and total soluble solids, varied within a narrower range across the sampled material.

#### 3.1.1. Coefficient of Variation

The coefficient of variation differed substantially among traits. Stem girth showed the highest coefficient of variation (95.10%), followed by stone weight (42.96%) and stone length (34.54%). Vitamin C and total soluble solids showed the lowest coefficients of variation, at 7.98% and 12.88%, respectively. These results indicate that vegetative architecture and stone-related traits contributed more strongly to quantitative variation than fruit biochemical traits.

#### 3.1.2. Province-Level Trait Comparison

One-way ANOVA revealed significant province-level differences for several quantitative traits (Table 2). Leaf length and leaf width differed highly significantly among provinces, with *p*-values of 1.35 × 10^−9^ and 1.33 × 10^−9^, respectively. Stone weight also differed highly significantly among provinces (*p* = 7.46 × 10^−5^), while significant differences were detected for total soluble solids (*p* = 2.21 × 10^−4^), stone length (*p* = 0.00112), east–west canopy spread (*p* = 0.00990), north–south canopy spread (*p* = 0.00988), and stem girth (*p* = 0.0386). In contrast, thorn size, fruit length, fruit width, stone width, acidity, and vitamin C did not differ significantly among provinces.

Province-level trends indicated that accessions from Khyber Pakhtunkhwa generally had greater canopy spread and stem girth. Punjab accessions tended to show larger fruit dimensions and heavier stones, whereas Sindh accessions were characterized by smaller leaves but relatively heavy stones. The province-by-cluster distribution (Supplementary Figure S2) confirms that all three provinces contributed to each cluster, so these regional trait trends did not translate into province-exclusive groups. Overall, these patterns suggest measurable regional differentiation in selected vegetative and stone-related traits, although not all traits showed significant geographic separation.

#### 3.1.3. Correlation Analysis

**Figure 3:**
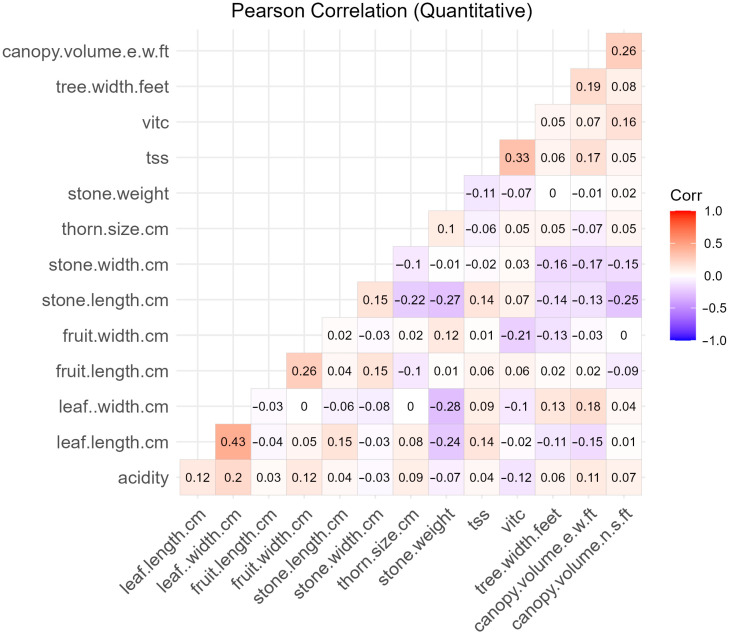
Pearson correlation (r) heatmap among 14 quantitative traits (*N* = 100). Positive correlations in red and negative in blue.

Pearson correlation analysis identified several moderate correlations among quantitative traits (Figure 3). Among the positive associations, the strongest was recorded between leaf length and leaf width (r = 0.43). Total soluble solids correlated with vitamin C content (r = 0.33), while fruit length was associated with fruit width (r = 0.26). A similar correlation was observed between canopy spread in the east–west and north–south directions (r = 0.26).

Negative correlations were detected between leaf width and total soluble solids (r = −0.28), stone length and total soluble solids (r = −0.27), and stone length and vitamin C (r = −0.25). These associations indicate that vegetative, stone, fruit, and biochemical traits varied in coordinated but generally moderate patterns, with some evidence of trade-offs between structural traits and fruit quality attributes.

#### 3.1.4. Principal Component Analysis (PCA)

Principal component analysis (PCA) of quantitative traits revealed that the first two principal components explained 26.38% of the total phenotypic variance, with PC1 accounting for 13.35% and PC2 for 13.03%. Because PC1 and PC2 together explained only 26.38% of the total quantitative trait variation, the PCA plot was interpreted as a low-dimensional visualization of major trait gradients rather than a complete representation of overall phenotypic diversity. PC1 was primarily associated with leaf width (0.51), leaf length (0.36), stone weight (0.34), east–west canopy spread (0.33), total soluble solids (0.31) and acidity (0.26). PC2 was driven mainly by stone length (0.51), followed by leaf length (0.36), canopy spread (north–south: 0.35; east–west: 0.30), stone weight (0.35) and stone width (0.28). The PCA biplot/scatterplot indicated partial overlap among accessions from different provinces, with only moderate separation along PC1 and PC2. The PCA biplot (Supplementary Figure S3) shows accessions from the three provinces overlapping along PC1 and PC2, indicating that leaf, canopy and stone traits structured the variation more than geographic origin.

#### 3.1.5. Cluster Analysis (Euclidean + Ward Method)

Hierarchical clustering using Euclidean distance and the Ward method separated the 100 accessions into three major clusters (Supplementary Figure S4). Each cluster contained accessions from more than one province, indicating that quantitative trait similarity did not follow a strictly geographic pattern.

Cluster 1 was characterized mainly by a broader canopy spread and larger leaf dimensions. Cluster 2 included accessions with relatively larger fruit dimensions, heavier stones, and intermediate vegetative traits. Cluster 3 comprised accessions with a more compact canopy architecture, smaller leaf dimensions, and variation in acidity and stone traits. Although some province-level tendencies were evident, accessions from Khyber Pakhtunkhwa, Punjab, and Sindh were intermixed across the three clusters. These results show that clustering was driven primarily by shared phenotypic attributes, particularly canopy architecture, leaf size, fruit size and stone morphology, rather than by province of origin alone.

### 3.2. Qualitative Trait Variation

Qualitative trait assessment revealed broad morphological diversity among the accessions. Tree growth habit ranged from erect to semi-erect and spreading, with the semi-erect habit being predominant. Leaf morphology was variable; acute and oval leaf shapes were most frequent, whereas round and oblong forms occurred less commonly. Leaf base was mostly obtuse, although cordate and intermediate forms were also observed. The upper leaf surface was predominantly dark green, with blackish-green and lighter green shades present in some accessions. Fruit-related descriptors showed pronounced variation. Fruit shape ranged from round, oval, ovate and oblong to falcate. Fruit surface was mostly plain, although warted and ridged surfaces were recorded in some Sindh accessions. Stone shape was predominantly round, but spindle-shaped stones were also observed in the accessions. Thorn traits also varied: caducous thorns were most common, while persistent thorns were observed in some accessions (Supplementary Figure S5). Taken together, these descriptors indicate substantial qualitative variation in tree habit, leaf morphology, thorn features, fruit appearance and stone form.

#### 3.2.1. Associations Among Qualitative Traits

**Figure 4:**
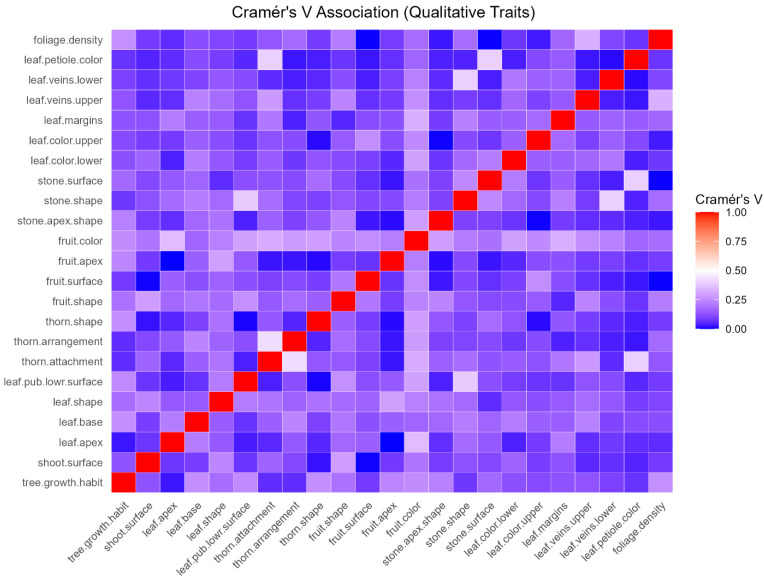
Cramér’s V association matrix for the qualitative traits scored in wild-growing/naturalized ber (*Ziziphus mauritiana* Lam.) accessions (N = 100). Each cell shows the pairwise association strength between two categorical descriptors; darker cells indicate stronger associations, as shown by the color scale bar (Cramér’s V: 0–1).

Associations among qualitative descriptors were examined using Cramér’s V and Spearman correlation matrices. The Cramér’s V heatmap showed moderate to strong associations among several biologically related traits, including fruit shape and fruit color (V = 0.40), leaf base and leaf pubescence (V = 0.39), and thorn arrangement and thorn attachment (V = 0.43) (Figure 4). Most other trait pairs showed weak associations, with Cramér’s V values below 0.20, indicating that many qualitative descriptors varied relatively independently.

Spearman rank correlations showed similar patterns (Supplementary Figure S6). Positive associations were observed among selected leaf and thorn descriptors, whereas relationships between fruit and leaf categories were generally weaker.

#### 3.2.2. Multiple Correspondence Analysis (MCA)

The MCA of qualitative traits accounted for 5.4% and 5.7% of total variance along the first two axes. The province scatterplot showed that Punjab had broader dispersion than Khyber Pakhtunkhwa and Sindh (Supplementary Figure S7). The categories of traits contributing most to Dimension 1 and Dimension 2 were thorn arrangement, thorn attachment, leaf pubescence and fruit color (Supplementary Table S3). These results suggest that these traits contributed most strongly to phenotypic variation and showed partial province-specific clustering, although overlap among populations was also evident.

#### 3.2.3. Cluster Analysis of Combined Quantitative and Qualitative Traits

**Figure 5:**
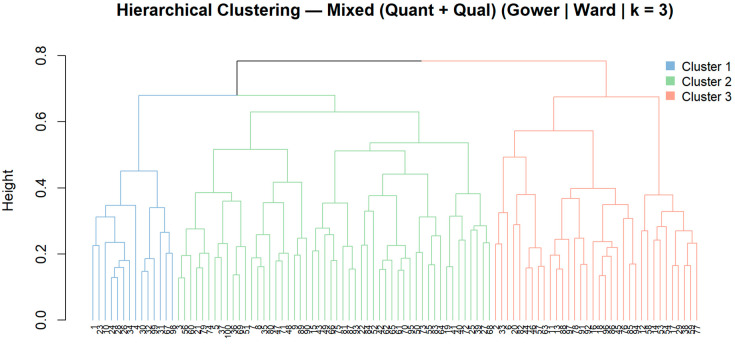
Hierarchical clustering of wild-growing/naturalized ber (*Ziziphus mauritiana* Lam.) accessions using mixed quantitative and qualitative traits. Clustering was performed using Gower distance and Ward linkage, and the dendrogram was cut into k = 3 clusters (color-coded branches), showing overall similarity patterns among accessions based on combined trait data.

Hierarchical clustering based on the Gower distance and the Ward method grouped the accessions into three main clusters (Figure 5). Cluster 1 was densely populated with accessions having spreading tree habit and persistent thorns. Cluster 2 grouped accessions with diverse fruit colors and shapes. Cluster 3 consisted of accessions with consistent leaf morphology, but varied stone traits. The dendrogram further indicated that, rather than displaying province-specific clustering, Khyber Pakhtunkhwa, Punjab and Sindh materials were intermixed across groups, suggesting that morphological differentiation was driven more by trait interplay than geographic provenance.

#### 3.2.4. Province Cluster Relationship

Contingency analysis was performed to evaluate the association of the provinces with cluster assignments. The cross-tabulation of the Province × Cluster table revealed that accessions from all three provinces (KP, PB, and SD) were distributed across the three major clusters derived from mixed-data hierarchical clustering. The largest proportion of the accessions fell into Cluster 3 for Khyber Pakhtunkhwa (23 accessions), and Punjab and Sindh had relatively equal distributions of the accessions in Clusters 2 and 3, respectively. The chi-square test of independence indicated a significant association between province and cluster membership (χ^2^ = 9.53, df = 4, and *p* = 0.049). Thus, cluster distribution was not entirely independent of province. However, the substantial overlap among provinces also indicates that accessions were not grouped exclusively by geographic origin, supporting a pattern of partial regional differentiation combined with broad phenotypic admixture.

#### 3.2.5. PERMANOVA of Phenotypic Trait Matrices

**Figure 6:**
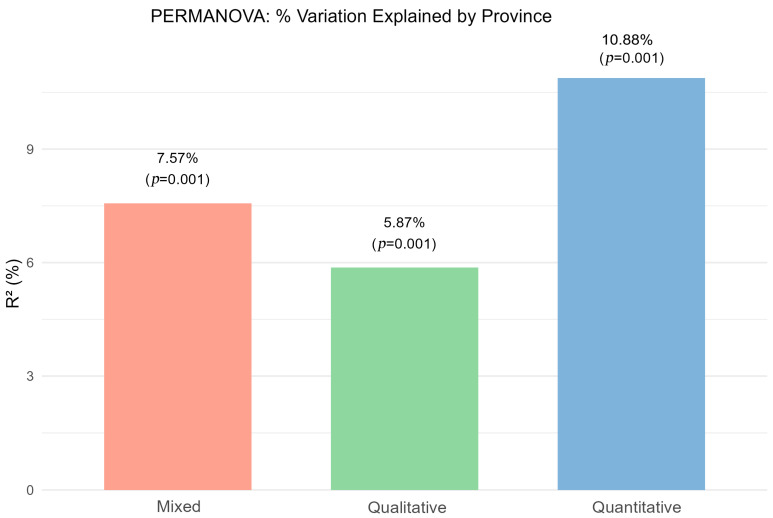
PERMANOVA results showing the percentage of variation (R^2^, %) explained by province of origin for three datasets: quantitative traits, qualitative traits, and mixed (quantitative + qualitative) traits in wild-growing/naturalized ber (*Ziziphus mauritiana* Lam.) accessions. Bars indicate the R^2^ (%) for each dataset, with associated *p*-values shown above bars.

PERMANOVA was used to test the effect of province on quantitative, qualitative, and combined phenotypic datasets (Figure 6). Province had a significant effect in all three analyses (*p* < 0.01), although the proportion of variance explained was modest. For quantitative traits, province explained 10.88% of the total multivariate variation (R^2^ = 0.109), indicating a clear geographic signal in the measured quantitative morphology. For qualitative traits, the provincial effect was smaller but still significant, accounting for 5.87% of variation (R^2^ = 0.0587), suggesting that categorical trait variation also tracked geographic origin. In the combined quantitative and qualitative dataset, province explained 7.57% of the total variation (R^2^ = 0.0757).

#### 3.2.6. Integrative Phenotypic Patterns

Integration of PCA, MCA, clustering, contingency analysis, and PERMANOVA revealed a consistent pattern of phenotypic differentiation. Among quantitative traits, leaf morphology, canopy dimensions, and stone-related characteristics were the principal contributors to accession variation. Among qualitative traits, thorn arrangement, thorn attachment, leaf pubescence, and fruit color were the dominant descriptors structuring diversity. Province-level analyses indicated significant but modest differentiation among Khyber Pakhtunkhwa, Punjab and Sindh. These differences were most evident for leaf, canopy, and stone traits, whereas biochemical traits such as total soluble solids and vitamin C showed comparatively limited variation. Overall, both quantitative descriptors, especially leaf, canopy, and stone traits, and qualitative descriptors, particularly thorn and fruit color traits, provided useful discriminating characteristics for assessing phenotypic diversity in the studied *Z. mauritiana* accessions.

### 3.3. Ploidy Level Determination

**Figure 7:**
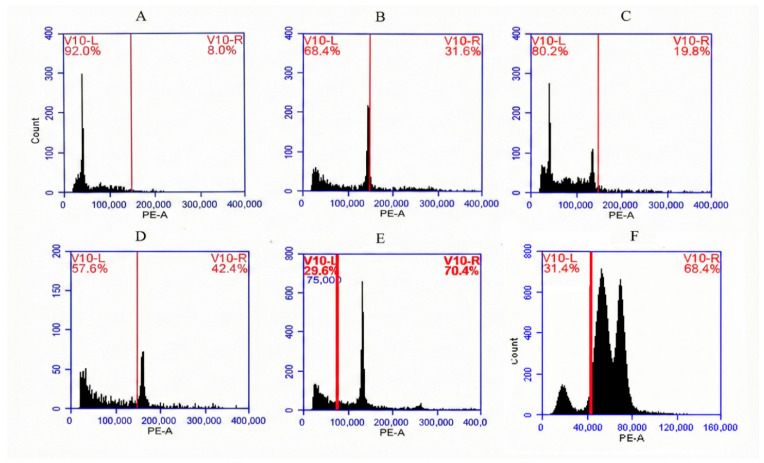
Representative flow cytometry PE-A histograms showing inferred cytotypes in *Ziziphus mauritiana*. (**A**) Diploid Dongzao reference standard (2x); (**B**,**C**) representative hexaploid (6x) accessions and mixed reference/sample run; (**D**) representative octoploid (8x) accession; (**E**,**F**) representative tetraploid (4x) accessions. Cytotype assignment was based on relative G0/G1 peak fluorescence compared with the diploid reference. The red vertical lines indicate the flow-cytometry gating/region boundaries used for fluorescence peak assessment.

Flow cytometry analysis revealed clear cytotype variation among the studied wild-growing/naturalized *Ziziphus mauritiana* accessions, with distinct fluorescence peaks corresponding to tetraploid (2*n* = 4x = 48), hexaploid (2*n* = 6x = 72) and octoploid (2*n* = 8x = 96) cytotypes (Figure 7). The diploid Dongzao (2*n* = 2x = 24) reference [9] showed a G0/G1 PE-A mean fluorescence of approximately 39,800. Based on this reference, tetraploid, hexaploid, and octoploid accessions were assigned to fluorescence ratio classes close to 2.0, 3.0, and 4.0, respectively. Representative hexaploid and octoploid accessions showed G0/G1 PE-A mean fluorescence values of approximately 116,700 and 160,800, corresponding to ratios of approximately 2.93 and 4.04 relative to the diploid reference, respectively. Representative tetraploid accessions showed G0/G1 PE-A mean fluorescence values of approximately 79,600, corresponding to a ratio of approximately 2.00 relative to the diploid Dongzao reference. Only histograms with distinct G0/G1 peaks, reproducible duplicate runs, and peak CV values below 5% were retained for cytotype assignment. Across the 60 accessions, 13 were tetraploid (21.7%), 28 hexaploid (46.7%) and 19 octoploid (31.7%); hexaploids were, therefore, the most frequent cytotype (Supplementary Table S4C). Clear hexaploid profiles were recorded in accessions from all three provinces, and both hexaploid and octoploid profiles were observed in Sindh accessions. Multiple cytotypes were, thus, present in wild-growing/naturalized *Z. mauritiana* from Pakistan.

### 3.4. SSR Marker Analysis

#### 3.4.1. Marker Performance and Within-Province Diversity

**Figure 8:**
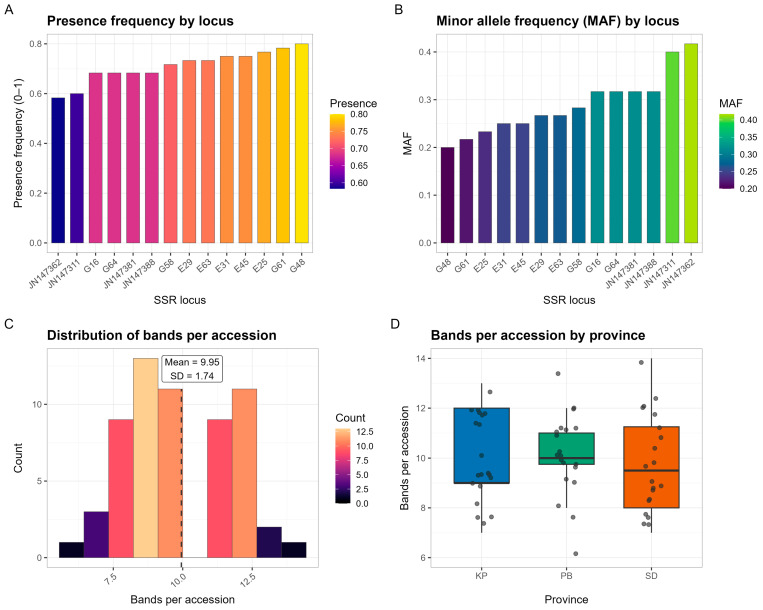
Locus informativeness and band richness across wild-growing/naturalized *Ziziphus mauritiana* accessions (KP, PB, and SD; N = 60, 14 SSR loci). (**A**) Band presence frequency per locus; (**B**) minor band frequency (MBF) per locus; (**C**) distribution of total bands per accession (mean = 9.95; SD = 1.74); (**D**) bands per accession by province. All loci were polymorphic (PPL = 100%).

Fourteen SSR primer pairs (Supplementary Table S2) were used to genotype 60 wild-growing/naturalized *Ziziphus mauritiana* accessions from Khyber Pakhtunkhwa, Punjab, and Sindh (Supplementary Figure S8). All loci were polymorphic (100% polymorphic loci). The band presence frequency per locus ranged from 0.59 to 0.80 (mean = 0.73; Figure 8A), and the minor band frequency (the frequency of the less common state under the binary scoring framework) ranged from 0.20 to 0.42 (mean = 0.29; Figure 8B), with no locus approaching monomorphism. The polymorphic information content, calculated from observed band frequencies under a binary marker framework, ranged from 0.33 to 0.49. The mean band richness per accession was similar across provinces (KP: 9.95 ± 1.76, PB: 10.10 ± 1.59, and SD: 9.80 ± 1.94; Figure 8C,D). The marker statistics are summarized in Supplementary Table S4A.

#### 3.4.2. Genetic Diversity and Population Structure

Nei’s gene diversity (H), estimated from the binary presence/absence matrix under a dominant-marker framework and interpreted as band pattern diversity, was similar among provinces: 0.515 (KP), 0.504 (PB), and 0.502 (SD), with an overall mean of 0.507 (Supplementary Table S4B). These values indicate comparable within-province band pattern diversity and little difference among provincial groups.

#### 3.4.3. Principal Coordinates Analysis (PCoA)

PCoA based on Jaccard distances explained 18.76% of the variation on axis 1 and 17.39% on axis 2 (Supplementary Figure S9); these low values indicate that the ordination summarizes broad structure rather than total diversity. Accessions from the three provinces were intermixed across the plot, with no province-level separation, consistent with weak geographic structuring.

#### 3.4.4. Unweighted Pair Group Method with Arithmetic Mean (UPGMA) Clustering

The UPGMA dendrogram based on Jaccard distances did not group accessions strictly by province (Supplementary Figure S10). Cluster 1 contained accessions from Khyber Pakhtunkhwa and Sindh; Cluster 2 was mainly Punjab with some Sindh accessions; and Cluster 3 contained accessions from all three provinces. Clusters, therefore, comprised accessions from multiple provinces, indicating weak geographic structuring in the SSR band pattern matrix.

#### 3.4.5. Genetic Differentiation Among Provinces

A one-factor PERMANOVA based on the Jaccard distance matrix showed no significant effect of province on SSR-based genetic composition (R^2^ = 0.0298, F = 0.876, and *p* = 0.6006). The test for homogeneity of multivariate dispersion was also non-significant (*p* = 0.8795), indicating that the PERMANOVA result was not influenced by unequal within-province dispersion. AMOVA further supported the absence of genetic structure among provinces, with the among-province variance component estimated as effectively zero, while nearly all variation was attributable to within-province differences. The fixation index was close to zero (Φ ≈ −0.011; *p* = 0.6005), confirming negligible genetic differentiation. Pairwise PERMANOVA comparisons were likewise non-significant for KP vs. PB (R^2^ = 0.027; *p* = 0.645), KP vs. SD (R^2^ = 0.0197; *p* = 0.645) and PB vs. SD (R^2^ = 0.0211; *p* = 0.645). Together, these results indicate that SSR-based genetic variation was distributed primarily within provinces rather than among provinces.

## 4. Discussion

This integrated study of 100 wild-growing/naturalized *Ziziphus mauritiana* accessions from Khyber Pakhtunkhwa (KP), Punjab (PB) and Sindh (SD) yielded three principal findings. The accessions exhibited substantial morphological variation dominated by canopy architecture, leaf dimensions, and stone-related traits, while fruit biochemical attributes varied within a comparatively narrow range. Flow cytometry revealed a multi-cytotype structure with hexaploids predominating and tetraploid and octoploid accessions also present, providing the first flow-cytometric cytotype assessment of *Z. mauritiana* in Pakistan. SSR analyses showed moderate within-province diversity but negligible differentiation among provinces. Taken together, these results frame Pakistani wild-growing/naturalized *Z. mauritiana* as a phenotypically diverse, polyploid and genetically connected germplasm resource whose value lies primarily within rather than between provinces.

The morphological variability detected here likely reflects both underlying genetic diversity and local adaptation. The greatest dispersion was observed for stem girth, stone weight, stone length, and canopy spread, identifying vegetative architecture and stone-related traits as the major axes of phenotypic divergence. These patterns are consistent with previous reports describing extensive morphological differentiation in *Z. mauritiana* and related *Ziziphus* [20,21] and with reports from India and China linking local environments to variation in tree habit and fruit size [11,22].

PERMANOVA attributed a small but significant share of variance to province, while most variation remained within provinces. KP accessions tended toward broader canopies, PB toward larger fruits and heavier stones, and SD toward smaller leaves with relatively heavy stones. These regional tendencies are consistent with environmentally influenced variation in vegetative and stone-related traits, though the relative contributions of genetic and environmental factors remain to be resolved. Comparable regional structuring of fruit and stone morphometrics has been reported previously [7,18].

Qualitative characteristics showed parallel variation, with PB more variable in fruit color and KP showing greater thorn persistence. Such variation may arise from a combination of natural and human selection, although the underlying drivers were not tested here; similar divergence in fruit apex, leaf pubescence, and thorn persistence has been associated with adaptation and domestication history in Indian jujube [6,23]. The reported high heritability and additive genetic control for fruit and stone weight further suggest that such characters can respond to phenotypic selection [24].

Correlation analysis revealed integrated trait modules, leaf dimensions covaried with canopy spread and fruit size, and TSSs were positively correlated with vitamin C, while size–quality trade-offs appeared between leaf width and TSSs and between stone length and both TSSs and vitamin C. Comparable positive associations between leaf and fruit size have been reported in Indian *Z. mauritiana* [24,25], and the coupling of flesh and stone biomass in Chinese jujube suggests that selection for fruit size induces correlated changes elsewhere [26,27]. The negative size–quality relationships are consistent with allocation trade-offs documented for *Z. mauritiana* under semi-arid conditions [1,6,28], so these characteristics should be treated as interconnected complexes in selection schemes [22].

Principal component analysis confirmed a multidimensional structure of phenotypic variation, with leaf morphology, canopy architecture, stone metrics, and fruit dimensions emerging as the principal discriminating traits, in agreement with prior germplasm studies [20,21,29]. Hierarchical clustering (Euclidean/Ward, k = 3) grouped accessions on the basis of trait combinations rather than provenance, with thorn characteristics and fruit color driving qualitative variation, consistent with cultivar-level studies in which these traits contributed to genotype discrimination [21,30] and with their recognized value for delineating genetic relationships within *Ziziphus* [31]. The province × cluster contingency analysis indicated a significant but weak association between origin and cluster membership, consistent with partial geographic structuring overlaid on broad intermixing [32]. These observations are in line with genome-scale evidence that domestication history has shaped a structured but connected diversity landscape in *Z. mauritiana* [10], and they support phenotypic selection that prioritizes within-province trait variation while drawing on modest province-level signals.

Flow cytometric profiling confirmed extensive polyploid diversity. Hexaploid (6x) accessions predominated, with tetraploid (4x) and octoploid (8x) cytotypes also detected, extending previous evidence that *Z. mauritiana* comprises a polyploid series beyond tetraploidy [5,9]. In other plant systems, polyploidy has been associated with abiotic stress tolerance [33,34,35]. The predominance of hexaploids in arid and semi-arid regions may suggest a possible adaptive advantage, but this cannot be confirmed from the present data. This study did not measure physiological traits, stress tolerance, water use efficiency, or fitness-related traits. Therefore, the adaptive significance of cytotype distribution is presented only as a hypothesis for future research, not as a conclusion. Further physiological and fitness comparisons among cytotypes are needed to test this possibility [36,37,38,39]. Nevertheless, the occurrence of multiple cytotypes expands the available germplasm base for future evaluation.

The weak phenotypic separation among provinces and the very low SSR differentiation are not contradictory. SSR markers mainly reflect neutral genetic variation, whereas quantitative traits can also be influenced by environmental plasticity and selection. Therefore, phenotypic differences among provinces should not be interpreted as direct evidence of neutral genetic differentiation. Instead, they may partly reflect environmental effects rather than clear population genetic structure.

SSR analyses provide a complementary view of diversity. All 14 loci were polymorphic, mean band richness was nearly identical across provinces, and Nei’s gene diversity was uniformly moderate, indicating that wild-growing/naturalized accessions retain a broad genetic base, consistent with the substantial SSR polymorphism reported for wild-growing/naturalized and cultivated *Ziziphus* taxa [9,13,40]. Comparable allelic richness has been documented in Chinese and Indian *Ziziphus* collections, where AMOVA consistently attributes most variance to within-population components [41,42], and the diversity values reported here fall within the range of other regional assessments [43]. Despite clear phenotypic differentiation, UPGMA, PCoA and AMOVA jointly indicated negligible geographic structuring, with a fixation index close to zero, consistent with the ~4% among-population variation reported in northwest India [10,42] and the high within-population diversity described in Pakistani *Z. nummularia* [43]. While domesticated *Z. mauritiana* and *Z. jujuba* show stronger structure at broader scales, reflecting cultivation history [9,44], the wild-growing/naturalized populations sampled here are weakly structured. This pattern is compatible with outcrossing, pollen and seed dispersal, and human-mediated movement of plant material [23,45], although these remain plausible rather than demonstrated mechanisms. Genome-scale studies similarly show that domestication has often proceeded against a backdrop of standing variation, with wild and cultivated forms connected by gene flow [10,46,47].

A few points should be considered when interpreting these results. First, although accessions were sampled across three provinces and multiple habitats, sampling was not formally stratified at the district scale, and denser sampling would further refine the diversity estimates. Second, the SSR data were scored from agarose gel profiles as a binary presence/absence matrix; in polyploid material, this captures multilocus band pattern diversity rather than dosage-resolved genotypes and has lower resolution than capillary electrophoresis, SNP genotyping, or genome-wide sequencing. Third, environmental and climatic variables for the collection sites were not included, so phenotypic variation could not be partitioned into genetic and environmental components. Future work using dosage-aware or sequence-based genotyping, georeferenced sampling, and common-garden or environmental analyses would address these points and allow more precise estimation of allele dosage and population structure.

Taken together, these results show that Pakistani wild-growing/naturalized *Z. mauritiana* represents a phenotypically diverse, cytotypically variable, but weakly structured gene pool, with most SSR variation occurring within rather than among provinces. This pattern is more similar to wild Pakistani *Ziziphus* resources than to strongly structured cultivated germplasm reported from broader regional collections [10,15,43,47]. Therefore, core collections and breeding panels should not be based only on province of origin. Instead, they should include accessions with distinct phenotypes, different ploidy levels, diverse ecological backgrounds, and representative molecular diversity. This approach would better capture the variation needed for conservation, parent selection, and future improvement of ber.

## 5. Conclusions

This study provides the first integrated phenotypic, cytotypic and SSR-based assessment of wild-growing/naturalized *Ziziphus mauritiana* in Pakistan. The germplasm forms a phenotypically diverse, predominantly hexaploid gene pool with moderate within-province genetic diversity and weak differentiation among provinces. Morphological variation is dominated by canopy, leaf and stone-related traits, whereas fruit biochemical attributes vary within a narrower range. Flow cytometry reveals tetraploid, hexaploid and octoploid cytotypes, with hexaploids most frequent. These findings establish a practical baseline for cytotype-aware germplasm conservation and core collection design in Pakistan. Future genomic studies using whole-genome sequencing, dosage-aware genotyping, or common-garden environmental trials are needed to clarify the functional significance of the different cytotypes and the genetic basis of trait variation.

## Figures and Tables

**Table 1:** Descriptive statistics for quantitative traits.

Trait	Min.	Max.	Mean	SD	CV (%)
Acidity	0.23	0.84	0.566	0.125	22.214
Canopy spread E–W (ft)	8.4	35.2	22.557	6.146	27.249
Canopy spread N–S (ft)	8.9	32.3	23.446	5.433	23.175
Fruit length (cm)	0.8	2.7	1.636	0.371	22.697
Fruit width (cm)	1.1	2.1	1.621	0.226	13.982
Leaf width (cm)	1	3.8	2.04	0.555	27.235
Leaf length (cm)	1.4	6.1	3.174	0.842	26.551
Stone length (cm)	0.4	2.1	0.861	0.297	34.544
Stone weight	0.13	0.83	0.402	0.172	42.956
Stone width (cm)	0.3	0.9	0.547	0.167	30.685
Thorn size (cm)	1	2.8	1.821	0.468	25.725
Stem girth feet	0.2	8	1.389	1.320	95.104
TSSs	5.46	9.43	7.528	0.969	12.878
Vitamin C	101.16	175.32	148.991	11.891	7.980

**Table 2:** One-way ANOVA *p*-values for morphological and fruit quality traits across Ber accessions.

Trait	*p*-Value (ANOVA)	Significant
Stem girth (ft)	0.0386	*
Leaf length (cm)	1.35 × 10^−9^	***
Leaf width (cm)	1.33 × 10^−9^	***
Thorn size (cm)	0.612	ns
Fruit length (cm)	0.283	ns
Fruit width (cm)	0.889	ns
Stone length (cm)	0.00112	**
Stone width (cm)	0.530	ns
Stone weight	7.46 × 10^−5^	***
TSSs	2.21 × 10^−4^	***
Acidity	0.142	ns
Vit-C	0.216	ns
Canopy spread E–W (ft)	0.00990	**
Canopy spread N–S (ft)	0.00988	**

Note: ns, not significant; * *p* < 0.05; ** *p* < 0.01; *** *p* < 0.001.

## Data Availability

The data are currently under curation for deposition in an open repository and will be released before publication. Until then, the data may be available from the corresponding author upon request.

## Supplementary Materials

The following supporting information can be downloaded at: https://www.mdpi.com/article/10.3390/plants15131974/s1. Figure S1. Representative wild-growing/naturalized *Ziziphus mauritiana* Lam. Figure S2. Distribution of the 14 quantitative traits across the three provinces (KP, PB, SD) in wild-growing/naturalized *Ziziphus mauritiana* accessions (N = 100), shown as box-and-whisker plots. Figure S3. Principal component analysis of the 14 quantitative traits: (A) variance explained per component, (B) top trait loadings on PC1 and PC2, (C) PCA scatterplot of accessions by province with 95% confidence ellipses. Figure S4. Hierarchical clustering of the 100 accessions based on quantitative traits (Euclidean distance, Ward.D2 linkage, k = 3); tip labels coloured by province. Figure S5. Category composition of the 23 qualitative traits by province (KP, PB, SD), shown as 100% stacked bar charts. Figure S6. Spearman rank-correlation matrix among the qualitative descriptors in wild-growing/naturalized *Ziziphus mauritiana* accessions. Figure S7. Multiple correspondence analysis of the qualitative traits: (A) individuals coloured by province with 95% confidence ellipses, (B) variance explained by the first six dimensions. Figure S8. Representative agarose-gel band profiles for selected SSR loci (E31, E45, G48, G58, G61, J81 & J88; 200 bp ladder) used to score band-presence patterns in the 60-accession SSR subset. Figure S9. Multivariate genetic structure of wild-growing/naturalized *Ziziphus mauritiana* accessions from SSR band-presence data: (A) PCoA on Jaccard distances, (B) DAPC; points coloured by province with 95% confidence ellipses. Figure S10. Circular UPGMA dendrogram of the 60 SSR-genotyped accessions based on Jaccard distances; tips coloured by province (KP, PB, SD). Table S1. Sampling metadata for the 100 wild-growing/naturalized *Ziziphus mauritiana* Lam. accessions: accession ID, province, district, broad habitat type, biological and management status, and collection period. Table S2. Primers used for nuclear simple sequence repeat (nSSR) amplification in this study. Supplementary Table S3. Categories of qualitative traits contributing most to the first MCA dimensions, with percentage contributions to Dimensions 1–5 and the combined Dimension 1–2 contribution. Table S4A. SSR marker performance and province-wise SSR band-pattern diversity based on the binary presence/absence matrix. Table S4B. Province-wise SSR band-pattern diversity. Table S4C. Cytotype frequencies among the 60 SSR-analysed *Z. mauritiana* accessions.

## Abbreviations

SSR, simple sequence repeat; MCA, multiple correspondence analysis; PERMANOVA, permutational multivariate analysis of variance; PCoA, principal coordinates analysis; PCA, principal component analysis; ANOVA, analysis of variance; AMOVA, analysis of molecular variance; UPGMA, unweighted pair group method with arithmetic mean; TSSs, total soluble solids; CV, coefficient of variation; MBF, minor band frequency; PIC, polymorphic information content; PPL, percentage of polymorphic loci; PI, propidium iodide; PCR, polymerase chain reaction; DNA, deoxyribonucleic acid; KP, Khyber Pakhtunkhwa; PB, Punjab; SD, Sindh.
