# Supplementary material for: Integrated Phenotypic, Cytotypic, and Microsatellite Diversity Analysis of Wild-Growing/Naturalized Ber (Ziziphus mauritiana Lam.) Across Pakistan: Implications for Germplasm Conservation and Breeding
Source: Plants (Basel). 2026 Jun 26;15(13):1974. doi: 10.3390/plants15131974 (PMC13364451; doi:10.3390/plants15131974)
Supplement: Supplementary file 1 [file plants-15-01974-s001.zip › plants-4340040-supplementary.pdf]

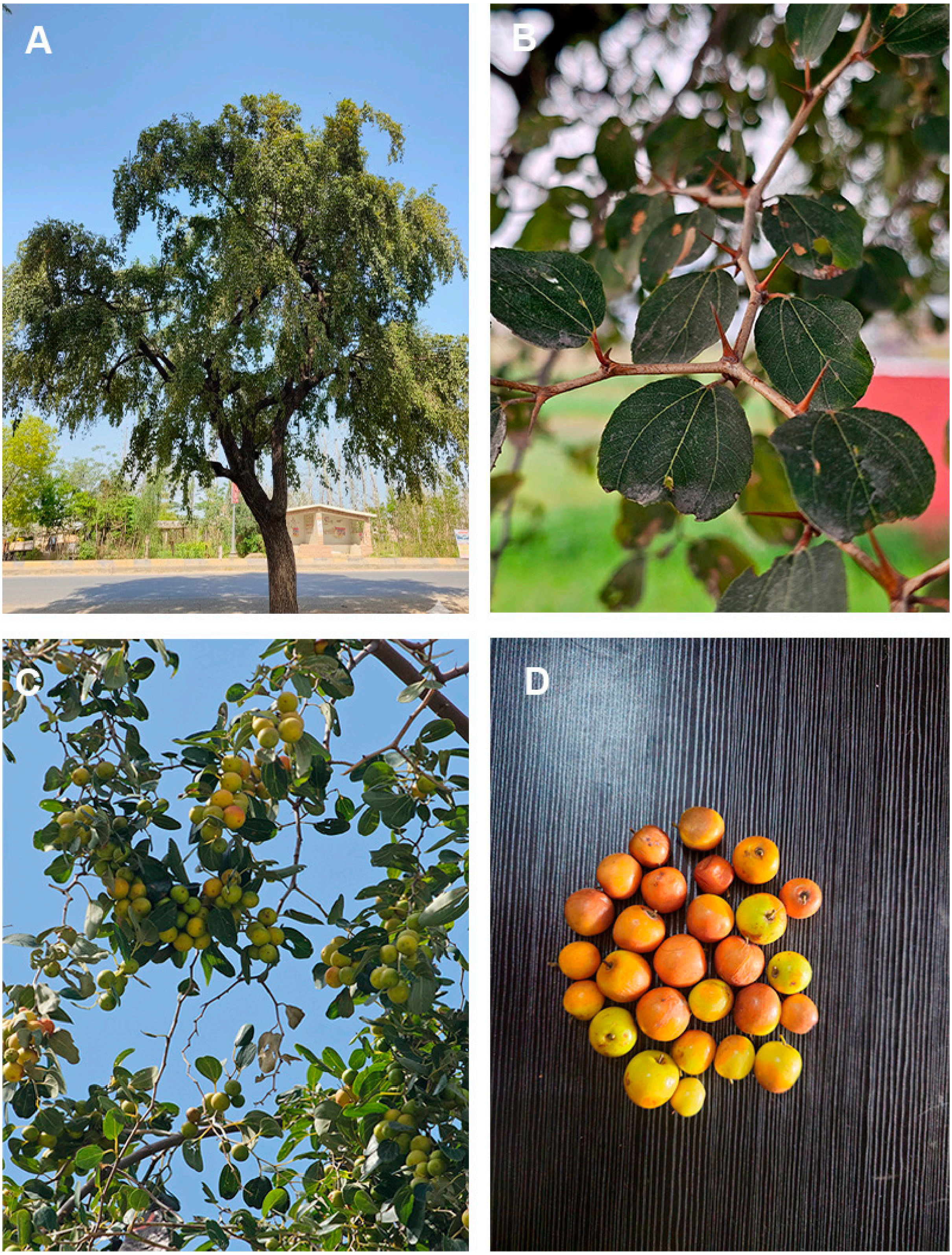

Supplementary Figure S1. Representative wild-growing/naturalized *Ziziphus mauritiana* Lam. in the field: (A) whole tree habit, (B) shoot with leaves and thorns, (C) fruiting branch, (D) harvested ripe fruits.

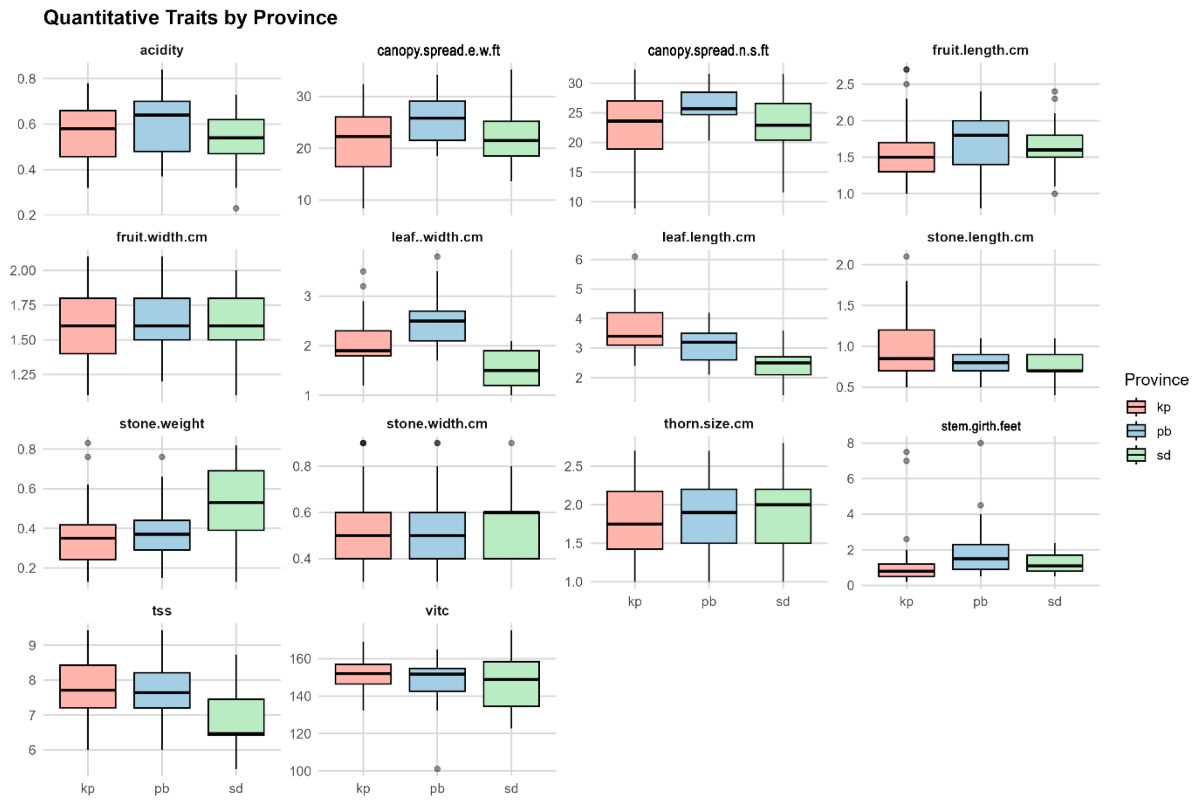

Supplementary Figure S2. Distribution of the 14 quantitative traits across the three provinces (KP, PB, SD) in wild-growing/naturalized *Ziziphus mauritiana* accessions (N = 100), shown as box-and-whisker plots.

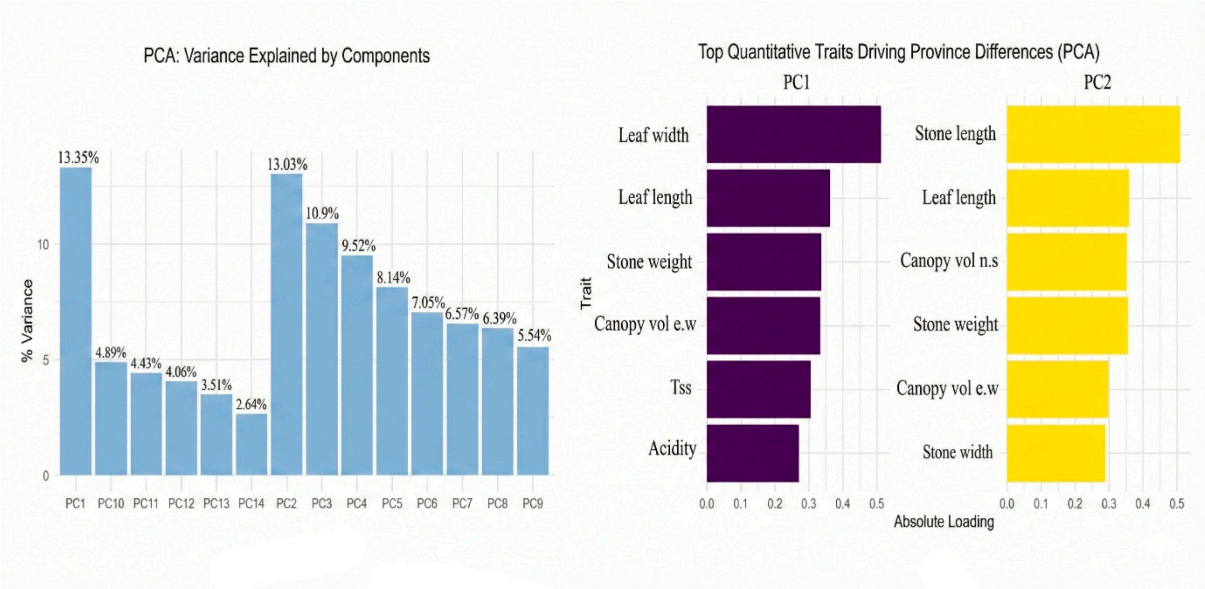

A **B**

C

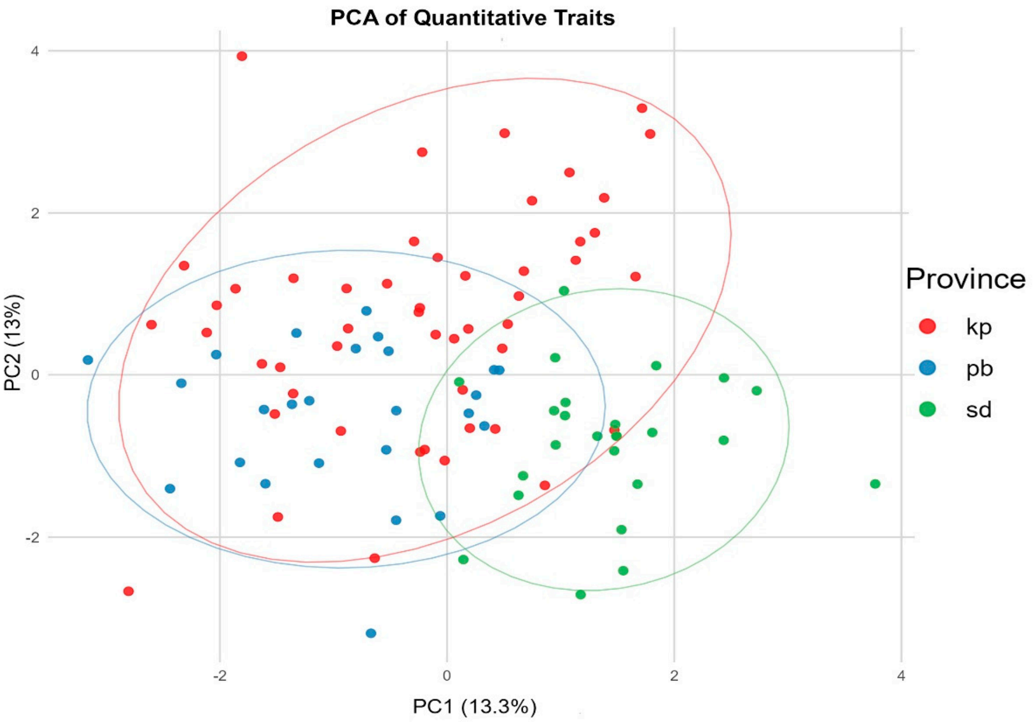

Supplementary Figure S3. Principal component analysis of the 14 quantitative traits: (A) variance explained per component, (B) top trait loadings on PC1 and PC2, (C) PCA scatterplot of accessions by province with 95% confidence ellipses.

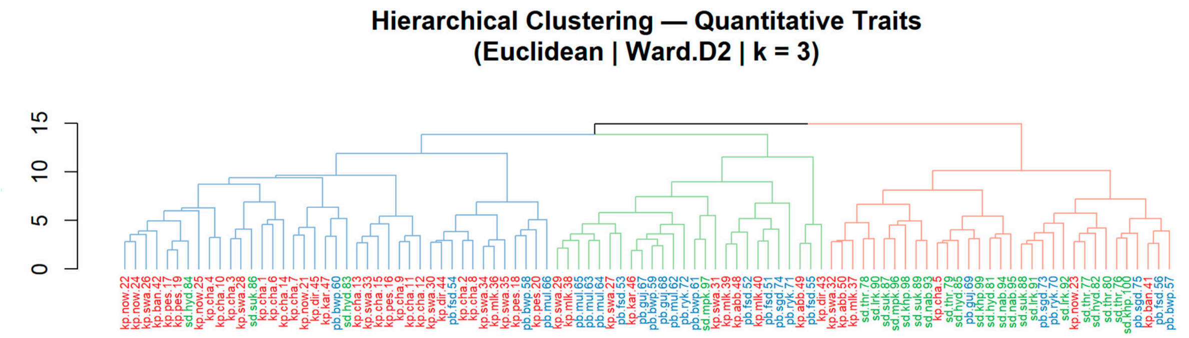

Supplementary Figure S4. Hierarchical clustering of the 100 accessions based on quantitative traits (Euclidean distance, Ward.D2 linkage, k = 3); tip labels coloured by province.

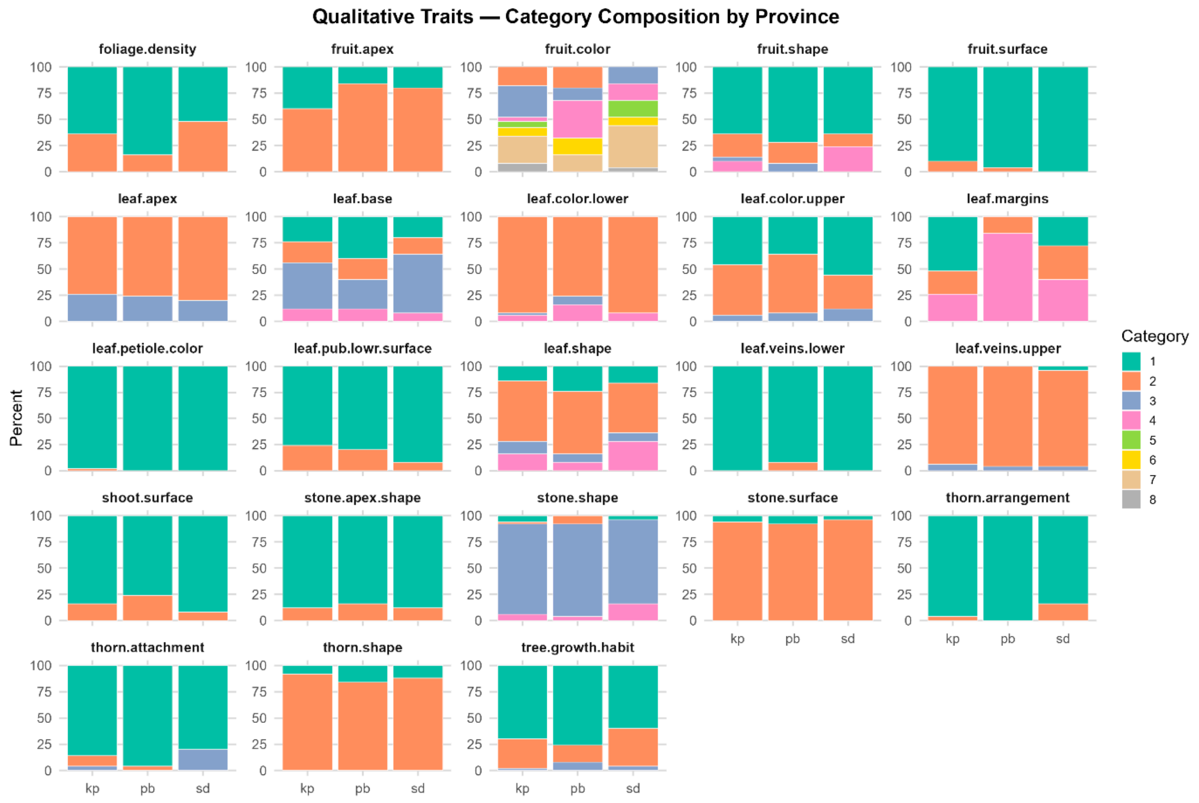

Supplementary Figure S5. Category composition of the 23 qualitative traits by province (KP, PB, SD), shown as 100% stacked bar charts.

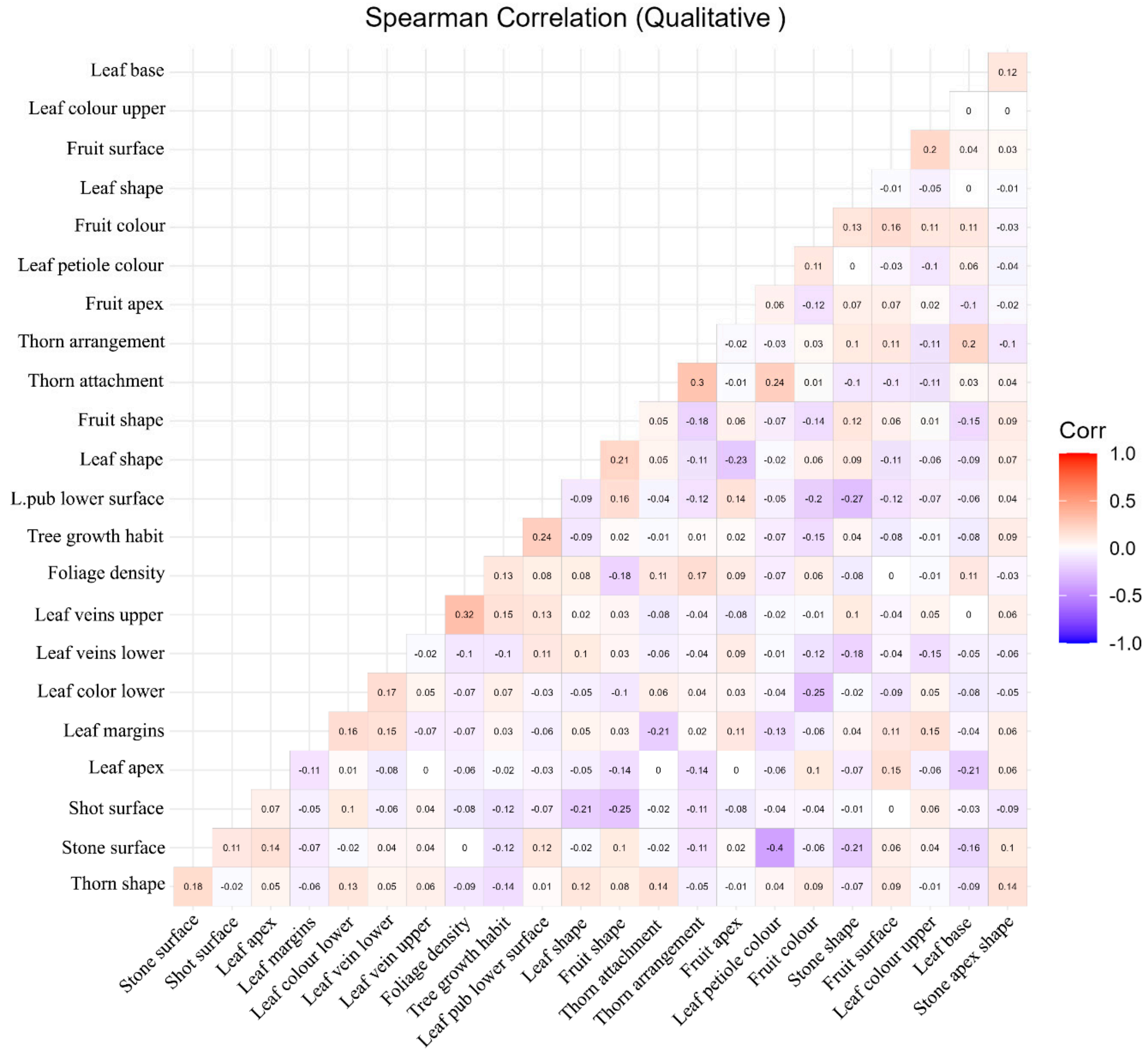

Supplementary Figure S6. Spearman rank-correlation matrix among the qualitative descriptors in wild-growing/naturalized *Ziziphus mauritiana* accessions.

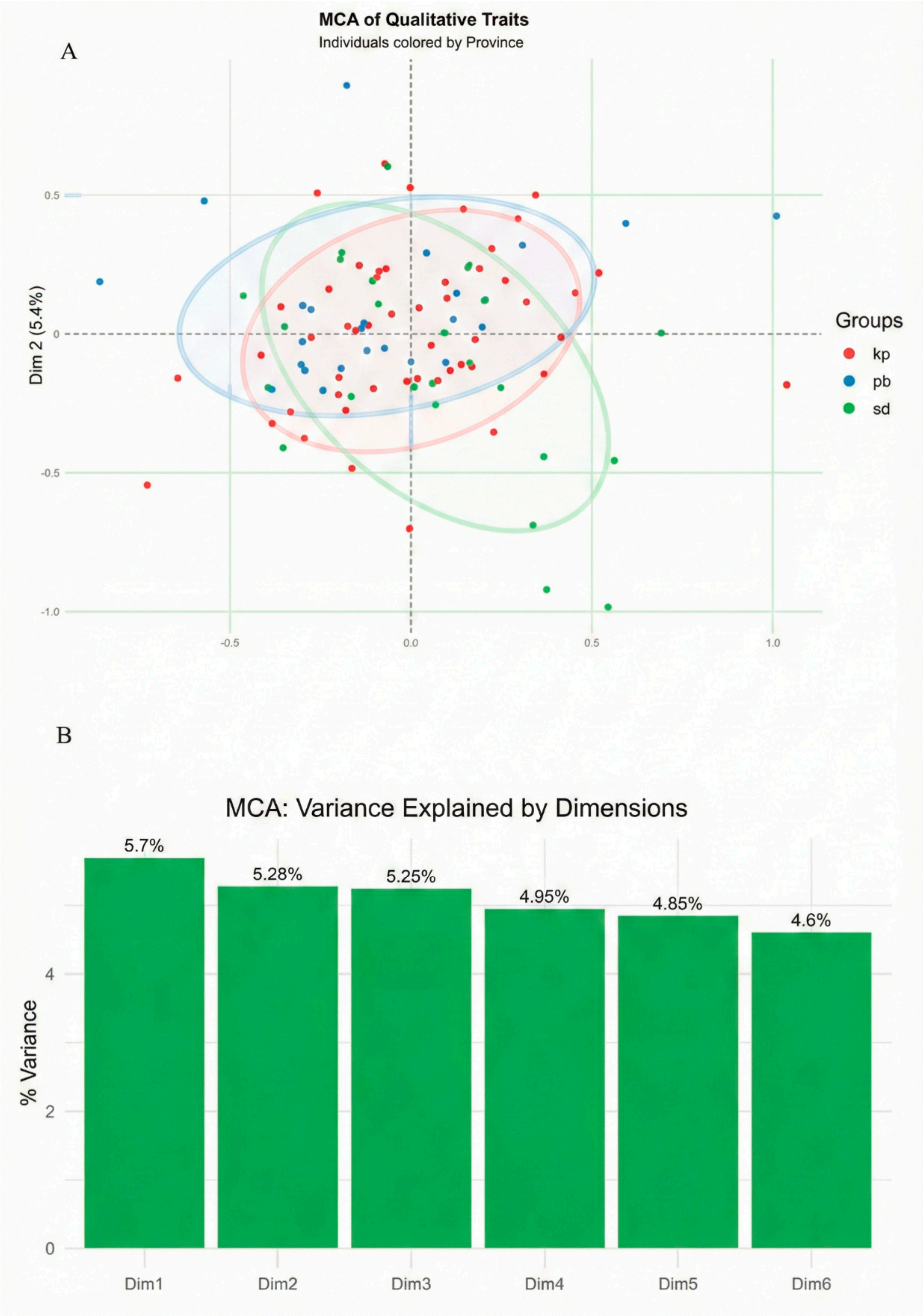

Supplementary Figure S7. Multiple correspondence analysis of the qualitative traits: (A) individuals coloured by province with 95% confidence ellipses, (B) variance explained by the first six dimensions.

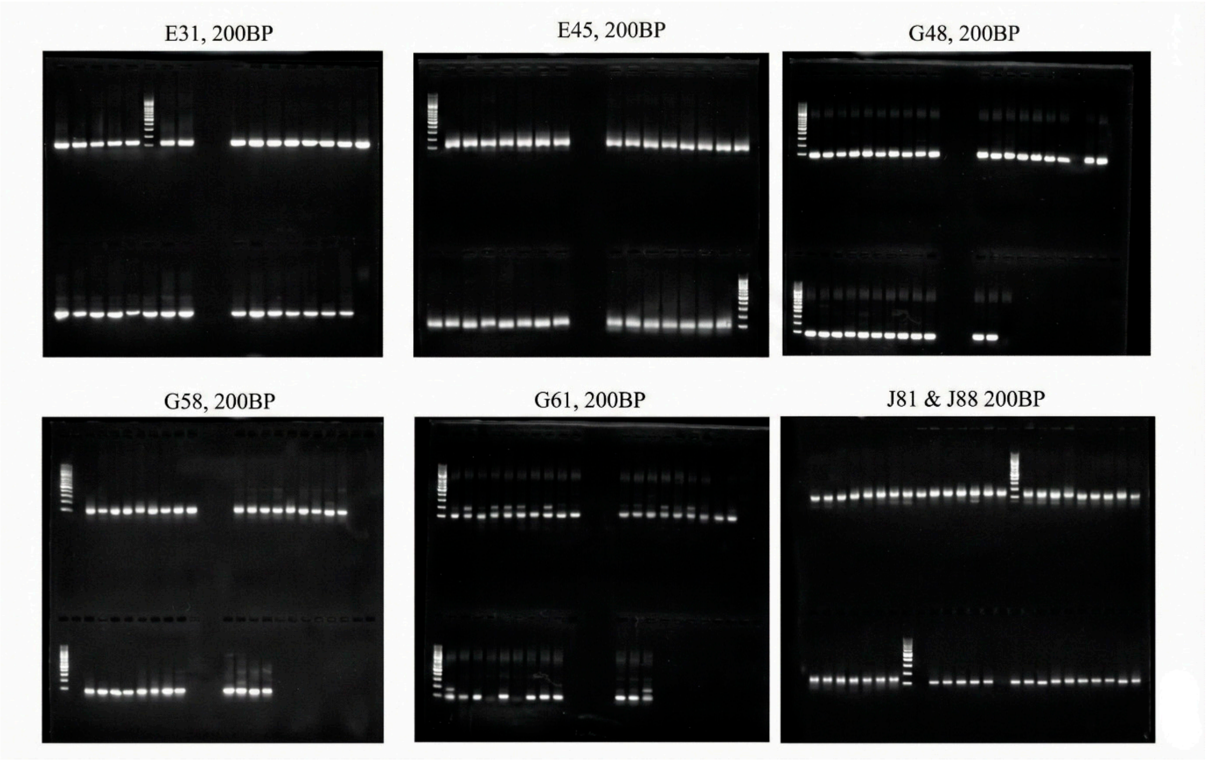

Supplementary Figure S8. Representative agarose-gel band profiles for selected SSR loci (E31, E45, G48, G58, G61, J81 & J88; 200 bp ladder) used to score band-presence patterns in the 60-accession SSR subset.

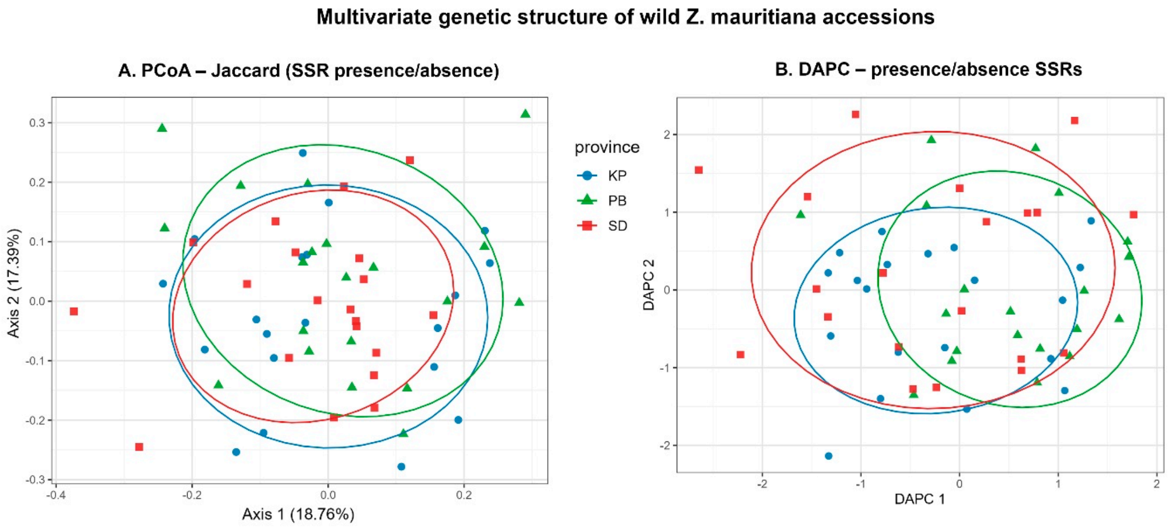

Supplementary Figure S9. Multivariate genetic structure of wild-growing/naturalized *Ziziphus mauritiana* accessions from SSR band-presence data: (A) PCoA on Jaccard distances, (B) DAPC; points coloured by province with 95% confidence ellipses.

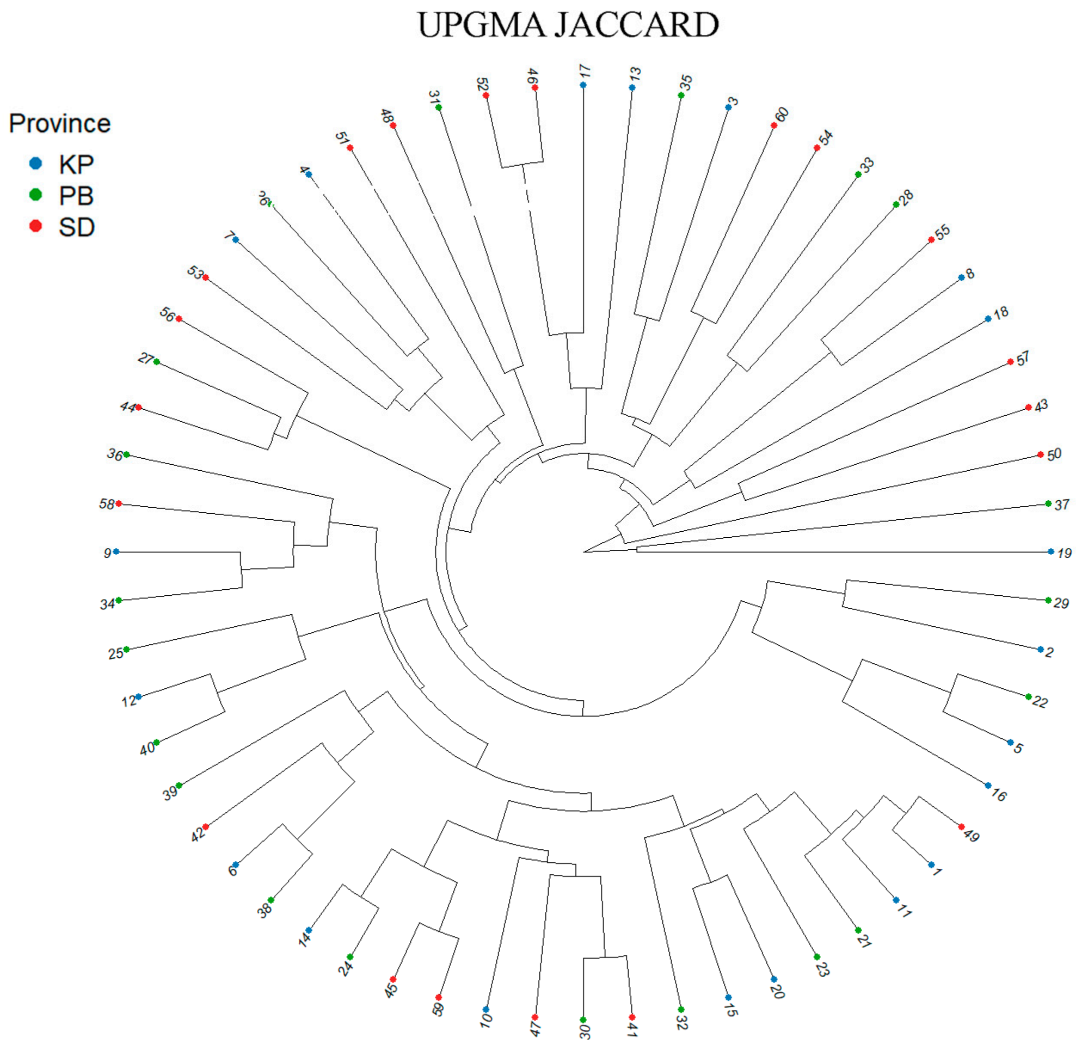

Supplementary Figure S10. Circular UPGMA dendrogram of the 60 SSR-genotyped accessions based on Jaccard distances; tips coloured by province (KP, PB, SD).

Supplementary Table S1. Sampling metadata for the 100 wild-growing/naturalized *Ziziphus mauritiana* Lam. accessions: accession ID, province, district, broad habitat type, biological and management status, and collection period.

Accession ID	Province code	Province	District code	District	Broad habitat type	Habitat / area descriptor	Biological status	Management status	Collection period
KP-CHA-001	KP	Khyber Pakhtunkhwa	CHA	Charsadda	Unmanaged roadside stand	Roadside and open unmanaged stand	Wild-growing/naturalized	Unmanaged self-sustaining;	2023–2025
KP-CHA-002	KP	Khyber Pakhtunkhwa	CHA	Charsadda	Unmanaged roadside stand	Roadside and open unmanaged stand	Wild-growing/naturalized	Unmanaged self-sustaining;	2023–2025

KP-CHA-003	KP	Khyber Pakhtunkhwa	CHA	Charsadda	Unmanaged roadside stand	Roadside and open unmanaged stand	Wild-growing/naturalized	Unmanaged self-sustaining;	2023–2025
KP-CHA-004	KP	Khyber Pakhtunkhwa	CHA	Charsadda	Unmanaged roadside stand	Roadside and open unmanaged stand	Wild-growing/naturalized	Unmanaged self-sustaining;	2023–2025
KP-CHA-005	KP	Khyber Pakhtunkhwa	CHA	Charsadda	Unmanaged roadside stand	Roadside and open unmanaged stand	Wild-growing/naturalized	Unmanaged self-sustaining;	2023–2025
KP-CHA-006	KP	Khyber Pakhtunkhwa	CHA	Charsadda	Unmanaged roadside stand	Roadside and open unmanaged stand	Wild-growing/naturalized	Unmanaged self-sustaining;	2023–2025
KP-CHA-007	KP	Khyber Pakhtunkhwa	CHA	Charsadda	Unmanaged roadside stand	Roadside and open unmanaged stand	Wild-growing/naturalized	Unmanaged self-sustaining;	2023–2025
KP-CHA-008	KP	Khyber Pakhtunkhwa	CHA	Charsadda	Unmanaged roadside stand	Roadside and open unmanaged stand	Wild-growing/naturalized	Unmanaged self-sustaining;	2023–2025
KP-CHA-009	KP	Khyber Pakhtunkhwa	CHA	Charsadda	Unmanaged roadside stand	Roadside and open unmanaged stand	Wild-growing/naturalized	Unmanaged self-sustaining;	2023–2025
KP-CHA-010	KP	Khyber Pakhtunkhwa	CHA	Charsadda	Unmanaged roadside stand	Roadside and open unmanaged stand	Wild-growing/naturalized	Unmanaged self-sustaining;	2023–2025
KP-CHA-011	KP	Khyber Pakhtunkhwa	CHA	Charsadda	Unmanaged roadside stand	Roadside and open unmanaged stand	Wild-growing/naturalized	Unmanaged self-sustaining;	2023–2025
KP-CHA-012	KP	Khyber Pakhtunkhwa	CHA	Charsadda	Unmanaged roadside stand	Roadside and open unmanaged stand	Wild-growing/naturalized	Unmanaged self-sustaining;	2023–2025
KP-CHA-013	KP	Khyber Pakhtunkhwa	CHA	Charsadda	Unmanaged roadside stand	Roadside and open unmanaged stand	Wild-growing/naturalized	Unmanaged self-sustaining;	2023–2025
KP-CHA-014	KP	Khyber Pakhtunkhwa	CHA	Charsadda	Unmanaged roadside stand	Roadside and open unmanaged stand	Wild-growing/naturalized	Unmanaged self-sustaining;	2023–2025
KP-CHA-015	KP	Khyber Pakhtunkhwa	CHA	Charsadda	Unmanaged roadside stand	Roadside and open unmanaged stand	Wild-growing/naturalized	Unmanaged self-sustaining;	2023–2025
KP-PES-001	KP	Khyber Pakhtunkhwa	PES	Peshawar	Unmanaged roadside stand	Roadside and peri-urban	Wild-growing/naturalized	Unmanaged self-sustaining;	2023–2025

						unmanaged stand			
KP-PES-002	KP	Khyber Pakhtunkhwa	PES	Peshawar	Unmanaged roadside stand	Roadside and peri-urban unmanaged stand	Wild-growing/naturalized	Unmanaged self-sustaining;	2023–2025
KP-PES-003	KP	Khyber Pakhtunkhwa	PES	Peshawar	Unmanaged roadside stand	Roadside and peri-urban unmanaged stand	Wild-growing/naturalized	Unmanaged self-sustaining;	2023–2025
KP-PES-004	KP	Khyber Pakhtunkhwa	PES	Peshawar	Unmanaged roadside stand	Roadside and peri-urban unmanaged stand	Wild-growing/naturalized	Unmanaged self-sustaining;	2023–2025
KP-PES-005	KP	Khyber Pakhtunkhwa	PES	Peshawar	Unmanaged roadside stand	Roadside and peri-urban unmanaged stand	Wild-growing/naturalized	Unmanaged self-sustaining;	2023–2025
KP-NOW-001	KP	Khyber Pakhtunkhwa	NOW	Nowshera	Unmanaged roadside stand	Roadside and open unmanaged stand	Wild-growing/naturalized	Unmanaged self-sustaining;	2023–2025
KP-NOW-002	KP	Khyber Pakhtunkhwa	NOW	Nowshera	Unmanaged roadside stand	Roadside and open unmanaged stand	Wild-growing/naturalized	Unmanaged self-sustaining;	2023–2025
KP-NOW-003	KP	Khyber Pakhtunkhwa	NOW	Nowshera	Unmanaged roadside stand	Roadside and open unmanaged stand	Wild-growing/naturalized	Unmanaged self-sustaining;	2023–2025
KP-NOW-004	KP	Khyber Pakhtunkhwa	NOW	Nowshera	Unmanaged roadside stand	Roadside and open unmanaged stand	Wild-growing/naturalized	Unmanaged self-sustaining;	2023–2025
KP-NOW-005	KP	Khyber Pakhtunkhwa	NOW	Nowshera	Unmanaged roadside stand	Roadside and open unmanaged stand	Wild-growing/naturalized	Unmanaged self-sustaining;	2023–2025
KP-SWA-001	KP	Khyber Pakhtunkhwa	SWA	Swat	Foothill / valley stand	Foothill and valley-side unmanaged stand	Wild-growing/naturalized	Unmanaged self-sustaining;	2023–2025
KP-SWA-002	KP	Khyber Pakhtunkhwa	SWA	Swat	Foothill / valley stand	Foothill and valley-side unmanaged stand	Wild-growing/naturalized	Unmanaged self-sustaining;	2023–2025
KP-SWA-003	KP	Khyber Pakhtunkhwa	SWA	Swat	Foothill / valley stand	Foothill and valley-side unmanaged stand	Wild-growing/naturalized	Unmanaged self-sustaining;	2023–2025
KP-SWA-004	KP	Khyber Pakhtunkhwa	SWA	Swat	Foothill / valley stand	Foothill and valley-side unmanaged stand	Wild-growing/naturalized	Unmanaged self-sustaining;	2023–2025

KP-SWA-005	KP	Khyber Pakhtunkhwa	SWA	Swat	Foothill / valley stand	Foothill and valley-side unmanaged stand	Wild-growing/naturalized	Unmanaged self-sustaining;	2023–2025
KP-SWA-006	KP	Khyber Pakhtunkhwa	SWA	Swat	Foothill / valley stand	Foothill and valley-side unmanaged stand	Wild-growing/naturalized	Unmanaged self-sustaining;	2023–2025
KP-SWA-007	KP	Khyber Pakhtunkhwa	SWA	Swat	Foothill / valley stand	Foothill and valley-side unmanaged stand	Wild-growing/naturalized	Unmanaged self-sustaining;	2023–2025
KP-SWA-008	KP	Khyber Pakhtunkhwa	SWA	Swat	Foothill / valley stand	Foothill and valley-side unmanaged stand	Wild-growing/naturalized	Unmanaged self-sustaining;	2023–2025
KP-SWA-009	KP	Khyber Pakhtunkhwa	SWA	Swat	Foothill / valley stand	Foothill and valley-side unmanaged stand	Wild-growing/naturalized	Unmanaged self-sustaining;	2023–2025
KP-SWA-010	KP	Khyber Pakhtunkhwa	SWA	Swat	Foothill / valley stand	Foothill and valley-side unmanaged stand	Wild-growing/naturalized	Unmanaged self-sustaining;	2023–2025
KP-MLK-001	KP	Khyber Pakhtunkhwa	MLK	Malakand	Foothill / valley stand	Foothill and valley-side unmanaged stand	Wild-growing/naturalized	Unmanaged self-sustaining;	2023–2025
KP-MLK-002	KP	Khyber Pakhtunkhwa	MLK	Malakand	Foothill / valley stand	Foothill and valley-side unmanaged stand	Wild-growing/naturalized	Unmanaged self-sustaining;	2023–2025
KP-MLK-003	KP	Khyber Pakhtunkhwa	MLK	Malakand	Foothill / valley stand	Foothill and valley-side unmanaged stand	Wild-growing/naturalized	Unmanaged self-sustaining;	2023–2025
KP-MLK-004	KP	Khyber Pakhtunkhwa	MLK	Malakand	Foothill / valley stand	Foothill and valley-side unmanaged stand	Wild-growing/naturalized	Unmanaged self-sustaining;	2023–2025
KP-MLK-005	KP	Khyber Pakhtunkhwa	MLK	Malakand	Foothill / valley stand	Foothill and valley-side unmanaged stand	Wild-growing/naturalized	Unmanaged self-sustaining;	2023–2025
KP-BAN-001	KP	Khyber Pakhtunkhwa	BAN	Bannu	Arid plain / rangeland	Open arid plain and rangeland-like unmanaged stand	Wild-growing/naturalized	Unmanaged self-sustaining;	2023–2025
KP-BAN-002	KP	Khyber Pakhtunkhwa	BAN	Bannu	Arid plain / rangeland	Open arid plain and rangeland-like unmanaged stand	Wild-growing/naturalized	Unmanaged self-sustaining;	2023–2025
KP-DIR-001	KP	Khyber Pakhtunkhwa	DIR	Dir	Foothill / valley stand	Foothill and valley-side	Wild-growing/naturalized	Unmanaged self-sustaining;	2023–2025

						unmanaged stand			
KP-DIR-002	KP	Khyber Pakhtunkhwa	DIR	Dir	Foothill / valley stand	Foothill and valley-side unmanaged stand	Wild-growing/naturalized	Unmanaged self-sustaining;	2023–2025
KP-DIR-003	KP	Khyber Pakhtunkhwa	DIR	Dir	Foothill / valley stand	Foothill and valley-side unmanaged stand	Wild-growing/naturalized	Unmanaged self-sustaining;	2023–2025
KP-KAR-001	KP	Khyber Pakhtunkhwa	KAR	Karak	Arid plain / rangeland	Dry plain and rangeland-like unmanaged stand	Wild-growing/naturalized	Unmanaged self-sustaining;	2023–2025
KP-KAR-002	KP	Khyber Pakhtunkhwa	KAR	Karak	Arid plain / rangeland	Dry plain and rangeland-like unmanaged stand	Wild-growing/naturalized	Unmanaged self-sustaining;	2023–2025
KP-ABB-001	KP	Khyber Pakhtunkhwa	ABB	Abbottabad	Foothill / valley stand	Foothill and upland unmanaged stand	Wild-growing/naturalized	Unmanaged self-sustaining;	2023–2025
KP-ABB-002	KP	Khyber Pakhtunkhwa	ABB	Abbottabad	Foothill / valley stand	Foothill and upland unmanaged stand	Wild-growing/naturalized	Unmanaged self-sustaining;	2023–2025
KP-ABB-003	KP	Khyber Pakhtunkhwa	ABB	Abbottabad	Foothill / valley stand	Foothill and upland unmanaged stand	Wild-growing/naturalized	Unmanaged self-sustaining;	2023–2025
PB-FSD-001	PB	Punjab	FSD	Faisalabad	Unmanaged roadside stand	Roadside and open unmanaged stand	Wild-growing/naturalized	Unmanaged self-sustaining;	2023–2025
PB-FSD-002	PB	Punjab	FSD	Faisalabad	Unmanaged roadside stand	Roadside and open unmanaged stand	Wild-growing/naturalized	Unmanaged self-sustaining;	2023–2025
PB-FSD-003	PB	Punjab	FSD	Faisalabad	Unmanaged roadside stand	Roadside and open unmanaged stand	Wild-growing/naturalized	Unmanaged self-sustaining;	2023–2025
PB-FSD-004	PB	Punjab	FSD	Faisalabad	Unmanaged roadside stand	Roadside and open unmanaged stand	Wild-growing/naturalized	Unmanaged self-sustaining;	2023–2025
PB-FSD-005	PB	Punjab	FSD	Faisalabad	Unmanaged roadside stand	Roadside and open unmanaged stand	Wild-growing/naturalized	Unmanaged self-sustaining;	2023–2025
PB-FSD-006	PB	Punjab	FSD	Faisalabad	Unmanaged roadside stand	Roadside and open unmanaged stand	Wild-growing/naturalized	Unmanaged self-sustaining;	2023–2025

PB-BWP-001	PB	Punjab	BWP	Bahawalpur	Arid plain / desert-margin stand	Arid plain and desert-margin unmanaged stand	Wild-growing/naturalized	Unmanaged self-sustaining;	2023–2025
PB-BWP-002	PB	Punjab	BWP	Bahawalpur	Arid plain / desert-margin stand	Arid plain and desert-margin unmanaged stand	Wild-growing/naturalized	Unmanaged self-sustaining;	2023–2025
PB-BWP-003	PB	Punjab	BWP	Bahawalpur	Arid plain / desert-margin stand	Arid plain and desert-margin unmanaged stand	Wild-growing/naturalized	Unmanaged self-sustaining;	2023–2025
PB-BWP-004	PB	Punjab	BWP	Bahawalpur	Arid plain / desert-margin stand	Arid plain and desert-margin unmanaged stand	Wild-growing/naturalized	Unmanaged self-sustaining;	2023–2025
PB-BWP-005	PB	Punjab	BWP	Bahawalpur	Arid plain / desert-margin stand	Arid plain and desert-margin unmanaged stand	Wild-growing/naturalized	Unmanaged self-sustaining;	2023–2025
PB-MUL-001	PB	Punjab	MUL	Multan	Arid plain / unmanaged roadside stand	Dry plain and roadside unmanaged stand	Wild-growing/naturalized	Unmanaged self-sustaining;	2023–2025
PB-MUL-002	PB	Punjab	MUL	Multan	Arid plain / unmanaged roadside stand	Dry plain and roadside unmanaged stand	Wild-growing/naturalized	Unmanaged self-sustaining;	2023–2025
PB-MUL-003	PB	Punjab	MUL	Multan	Arid plain / unmanaged roadside stand	Dry plain and roadside unmanaged stand	Wild-growing/naturalized	Unmanaged self-sustaining;	2023–2025
PB-MUL-004	PB	Punjab	MUL	Multan	Arid plain / unmanaged roadside stand	Dry plain and roadside unmanaged stand	Wild-growing/naturalized	Unmanaged self-sustaining;	2023–2025
PB-MUL-005	PB	Punjab	MUL	Multan	Arid plain / unmanaged roadside stand	Dry plain and roadside unmanaged stand	Wild-growing/naturalized	Unmanaged self-sustaining;	2023–2025
PB-GUJ-001	PB	Punjab	GUJ	Gujranwala	Unmanaged roadside stand	Roadside and open unmanaged stand	Wild-growing/naturalized	Unmanaged self-sustaining;	2023–2025
PB-GUJ-002	PB	Punjab	GUJ	Gujranwala	Unmanaged roadside stand	Roadside and open unmanaged stand	Wild-growing/naturalized	Unmanaged self-sustaining;	2023–2025
PB-GUJ-003	PB	Punjab	GUJ	Gujranwala	Unmanaged roadside stand	Roadside and open unmanaged stand	Wild-growing/naturalized	Unmanaged self-sustaining;	2023–2025
PB-RYK-001	PB	Punjab	RYK	Rahim Yar Khan	Arid plain / desert-margin stand	Arid plain and desert-margin	Wild-growing/naturalized	Unmanaged self-sustaining;	2023–2025

						unmanaged stand			
PB-RYK-002	PB	Punjab	RYK	Rahim Yar Khan	Arid plain / desert-margin stand	Arid plain and desert-margin unmanaged stand	Wild-growing/naturalized	Unmanaged self-sustaining;	2023–2025
PB-RYK-003	PB	Punjab	RYK	Rahim Yar Khan	Arid plain / desert-margin stand	Arid plain and desert-margin unmanaged stand	Wild-growing/naturalized	Unmanaged self-sustaining;	2023–2025
PB-SGD-001	PB	Punjab	SGD	Sargodha	Unmanaged roadside stand	Roadside and open unmanaged stand	Wild-growing/naturalized	Unmanaged self-sustaining;	2023–2025
PB-SGD-002	PB	Punjab	SGD	Sargodha	Unmanaged roadside stand	Roadside and open unmanaged stand	Wild-growing/naturalized	Unmanaged self-sustaining;	2023–2025
PB-SGD-003	PB	Punjab	SGD	Sargodha	Unmanaged roadside stand	Roadside and open unmanaged stand	Wild-growing/naturalized	Unmanaged self-sustaining;	2023–2025
SD-THR-001	SD	Sindh	THR	Tharparkar	Desert-margin / rangeland	Desert-margin and rangeland-like unmanaged stand	Wild-growing/naturalized	Unmanaged self-sustaining;	2023–2025
SD-THR-002	SD	Sindh	THR	Tharparkar	Desert-margin / rangeland	Desert-margin and rangeland-like unmanaged stand	Wild-growing/naturalized	Unmanaged self-sustaining;	2023–2025
SD-THR-003	SD	Sindh	THR	Tharparkar	Desert-margin / rangeland	Desert-margin and rangeland-like unmanaged stand	Wild-growing/naturalized	Unmanaged self-sustaining;	2023–2025
SD-THR-004	SD	Sindh	THR	Tharparkar	Desert-margin / rangeland	Desert-margin and rangeland-like unmanaged stand	Wild-growing/naturalized	Unmanaged self-sustaining;	2023–2025
SD-THR-005	SD	Sindh	THR	Tharparkar	Desert-margin / rangeland	Desert-margin and rangeland-like unmanaged stand	Wild-growing/naturalized	Unmanaged self-sustaining;	2023–2025
SD-HYD-001	SD	Sindh	HYD	Hyderabad	Arid plain / unmanaged roadside stand	Dry plain and roadside unmanaged stand	Wild-growing/naturalized	Unmanaged self-sustaining;	2023–2025
SD-HYD-002	SD	Sindh	HYD	Hyderabad	Arid plain / unmanaged roadside stand	Dry plain and roadside unmanaged stand	Wild-growing/naturalized	Unmanaged self-sustaining;	2023–2025
SD-HYD-003	SD	Sindh	HYD	Hyderabad	Arid plain / unmanaged roadside stand	Dry plain and roadside unmanaged stand	Wild-growing/naturalized	Unmanaged self-sustaining;	2023–2025

SD-HYD-004	SD	Sindh	HYD	Hyderabad	Arid plain / unmanaged roadside stand	Dry plain and roadside unmanaged stand	Wild-growing/naturalized	Unmanaged self-sustaining;	2023–2025
SD-HYD-005	SD	Sindh	HYD	Hyderabad	Arid plain / unmanaged roadside stand	Dry plain and roadside unmanaged stand	Wild-growing/naturalized	Unmanaged self-sustaining;	2023–2025
SD-SUK-001	SD	Sindh	SUK	Sukkur	Arid plain / unmanaged roadside stand	Dry plain and roadside unmanaged stand	Wild-growing/naturalized	Unmanaged self-sustaining;	2023–2025
SD-SUK-002	SD	Sindh	SUK	Sukkur	Arid plain / unmanaged roadside stand	Dry plain and roadside unmanaged stand	Wild-growing/naturalized	Unmanaged self-sustaining;	2023–2025
SD-SUK-003	SD	Sindh	SUK	Sukkur	Arid plain / unmanaged roadside stand	Dry plain and roadside unmanaged stand	Wild-growing/naturalized	Unmanaged self-sustaining;	2023–2025
SD-SUK-004	SD	Sindh	SUK	Sukkur	Arid plain / unmanaged roadside stand	Dry plain and roadside unmanaged stand	Wild-growing/naturalized	Unmanaged self-sustaining;	2023–2025
SD-LRK-001	SD	Sindh	LRK	Larkana	Arid plain / unmanaged roadside stand	Dry plain and roadside unmanaged stand	Wild-growing/naturalized	Unmanaged self-sustaining;	2023–2025
SD-LRK-002	SD	Sindh	LRK	Larkana	Arid plain / unmanaged roadside stand	Dry plain and roadside unmanaged stand	Wild-growing/naturalized	Unmanaged self-sustaining;	2023–2025
SD-LRK-003	SD	Sindh	LRK	Larkana	Arid plain / unmanaged roadside stand	Dry plain and roadside unmanaged stand	Wild-growing/naturalized	Unmanaged self-sustaining;	2023–2025
SD-NAB-001	SD	Sindh	NAB	Nawabshah	Arid plain / unmanaged roadside stand	Dry plain and roadside unmanaged stand	Wild-growing/naturalized	Unmanaged self-sustaining;	2023–2025
SD-NAB-002	SD	Sindh	NAB	Nawabshah	Arid plain / unmanaged roadside stand	Dry plain and roadside unmanaged stand	Wild-growing/naturalized	Unmanaged self-sustaining;	2023–2025
SD-NAB-003	SD	Sindh	NAB	Nawabshah	Arid plain / unmanaged roadside stand	Dry plain and roadside unmanaged stand	Wild-growing/naturalized	Unmanaged self-sustaining;	2023–2025
SD-MPK-001	SD	Sindh	MPK	Mirpur Khas	Arid plain / unmanaged roadside stand	Dry plain and roadside unmanaged stand	Wild-growing/naturalized	Unmanaged self-sustaining;	2023–2025

SD-MPK-002	SD	Sindh	MPK	Mirpur Khas	Arid plain / unmanaged roadside stand	Dry plain and roadside unmanaged stand	Wild-growing/naturalized	Unmanaged self-sustaining;	2023–2025
SD-KHP-001	SD	Sindh	KHP	Khairpur	Arid plain / rangeland	Dry plain and rangeland-like unmanaged stand	Wild-growing/naturalized	Unmanaged self-sustaining;	2023–2025
SD-KHP-002	SD	Sindh	KHP	Khairpur	Arid plain / rangeland	Dry plain and rangeland-like unmanaged stand	Wild-growing/naturalized	Unmanaged self-sustaining;	2023–2025
SD-KHP-003	SD	Sindh	KHP	Khairpur	Arid plain / rangeland	Dry plain and rangeland-like unmanaged stand	Wild-growing/naturalized	Unmanaged self-sustaining;	2023–2025

Supplementary Table S2. Primers used for nuclear simple sequence repeat (nSSR) amplification in this study.

nSSR loci	Forward primer (5'-3')	Reverse primer (5'-3')	Repeats motif
E25	TTCTTCAGTTCAGAGGGAGCG	GTTTGCCTTTCTGCTTCTGTGC	(AG)13
E29	CAGCGTCATCTCATTCACATTTTC	CCCCTCATGTGCTTGAGTGT	(AG)12
E31	CAACGAAAGGCAGCTGACAAGG	GCAACGCCAGGGAGATAG	(GA)10
E45	TCTTTCGGCCACTCCATTAGGA	CCCACCTTACCGAACATAACCC	(AG)10
E63	GATACGGATACGGACGGAGAA	CGCAAACAAAGGAAACGAA	(GA)7
G16	ATGGGGATTACAGCTCACTTTTG	TGGGTGGTAGGAAAGTAGCTAT	(TC)16
G48	TTGGATGGTAACAAATCAGCTA	TGCTGCTGTCCATCTCCTT	(CT)12
G58	CTCTTCCTTGTTGGTGCTGGG	CGAGGTGTGTGCTAGTCCTT	(AC)10
G61	GACTACATCTCCCTCGACCACC	GGGTTGAAATGCGAGGT	(AG)9
G64	CAATCACGTGGCATGGGTAC	GGGCAAAATTCCAAACAAAC	(TCAGGG)6
JN147311	CTCATTGCTCCCAAGTGCAGG	ACCCCACCAAAACAAAGGC	(CT)20

JN147388	CCATTGCTCCCAAGTGCAGG	ACCCCACCAAAACAAAGGC	(CT)29
JN147381	AGGTGCCAACACGAGCCCA	TGCGCCACGCGGCGATGA	(GA)15
JN147362	TCGAGACGGTTGGAAGTGGGTTGG	GTTCCTCACCAACACTCACTC	(AG)19

Supplementary Table S3. Categories of qualitative traits contributing most to the first MCA dimensions, with percentage contributions to Dimensions 1–5 and the combined Dimension 1–2 contribution.

Category	Dim 1	Dim 2	Dim 3	Dim 4	Dim 5	Combined_Dim1_2
thorn.attachment_3	8.7847747	7.610134	0.203768	6.014103	2.273674	16.39491
thorn.arrangement_2	0.3621861	13.68251	0.948812	4.784058	0.021384	14.04469
leaf.pub.lowr.surface_2	4.6981611	8.706213	0.000127	0.297416	0.019816	13.40437
fruit.color_5	4.5374276	5.457051	0.360822	5.543785	0.275412	9.994479
stone.shape_2	9.3350789	0.637273	0.014583	0.171004	3.019708	9.972352
leaf.base_3	0.0658088	6.938269	0.465162	0.050237	0.022827	7.004078
stone.surface_1	6.4107819	0.458067	5.074517	2.090996	0.307064	6.868849
leaf.base_2	0.7246013	5.386098	4.59661	0.757286	0.058088	6.1107
fruit.color_2	2.8486365	2.954271	1.505017	2.473847	0.573386	5.802907
leaf.veins.upper_3	2.333E-06	5.692901	4.909368	0.304952	0.110317	5.692903
leaf.margins_2	5.1056384	0.542734	0.003193	0.180237	2.322636	5.648372
fruit.color_7	3.1460162	2.088534	0.023171	4.66564	1.126633	5.23455

Table S4A. SSR marker performance and province-wise SSR band-pattern diversity based on the binary presence/absence matrix.

A. Overall SSR marker performance

No. of SSR accessions	No. of SSR primer pairs	Polymorphic loci / primer pairs (%)	Presence frequency	Minor band frequency	Binary PIC
60	14	100	0.59–0.80;<br>mean = 0.73	0.20–0.42;<br>mean = 0.29	0.33–0.49

Table S4B. Province-wise SSR band-pattern diversity

**Province**	**Number of SSR accessions**	**Mean band richness per accession**	**Nei's binary band-frequency diversity (H)**
Khyber Pakhtunkhwa	20	9.95 ± 1.76	0.515
Punjab	20	10.10 ± 1.59	0.504
Sindh	20	9.80 ± 1.94	0.502
**Overall / mean**	**60**	**9.95 ± 1.74**	**0.507**

All 14 primer pairs were polymorphic. MAF, minor band frequency; PIC, polymorphic information content, calculated as 2pq from observed binary band frequencies. Band richness is the mean number of bands per accession (± SD). Nei's H is interpreted as band-pattern diversity under a dominant-marker framework.

Table S4C. Cytotype frequencies among the 60 SSR-analysed Z. mauritiana accessions.

**Cytotype**	**Number of accessions**	**Percentage (%)**
Tetraploid (4x)	13	21.7
Hexaploid (6x)	28	46.7
Octoploid (8x)	19	31.7
**Total**	**60**	**100**

Ploidy was determined by flow cytometry for all 60 accessions in the SSR subset.
